# Distinct myeloid-derived suppressor cell populations in human glioblastoma

**DOI:** 10.1126/science.abm5214

**Published:** 2025-01-17

**Authors:** Christina Jackson, Christopher Cherry, Sadhana Bom, Arbor G. Dykema, Rulin Wang, Elizabeth Thompson, Ming Zhang, Runzhe Li, Zhicheng Ji, Wenpin Hou, Wentao Zhan, Hao Zhang, John Choi, Ajay Vaghasia, Landon Hansen, William Wang, Brandon Bergsneider, Kate M. Jones, Fausto Rodriguez, Jon Weingart, Calixto-Hope Lucas, Jonathan Powell, Jennifer Elisseeff, Srinivasan Yegnasubramanian, Michael Lim, Chetan Bettegowda, Hongkai Ji, Drew Pardoll

**Affiliations:** 1 Department of Neurosurgery, Perelman School of Medicine, University of Pennsylvania, Philadelphia, PA, USA; 2 Institute for Immunology, Perelman School of Medicine, University of Pennsylvania, Philadelphia, PA, USA; 3 The Mark Foundation Center for Immunotherapy, Immune Signaling, and Radiation, University of Pennsylvania, Philadelphia, PA, USA; 4 Translational Tissue Engineering Center, Wilmer Eye Institute, and Department of Biomedical Engineering, Johns Hopkins University School of Medicine, Baltimore, MD, USA; 5 Bloomberg~Kimmel Institute for Cancer Immunotherapy, Baltimore, MD, USA; 6 Mark Center for Advanced Genomics and Imaging at the Johns Hopkins University, Baltimore, MD, USA; 7 The Sidney Kimmel Comprehensive Cancer Center at Johns Hopkins University, Baltimore, MD, USA; 8 Department of Biostatistics, Johns Hopkins Bloomberg School of Public Health, Baltimore, MD, USA; 9 Department of Molecular Microbiology and Immunology, Johns Hopkins Bloomberg School of Public Health, Baltimore, MD, USA; 10 Department of Neurosurgery, Johns Hopkins University School of Medicine, Baltimore, MD, USA; 11 Department of Neurosurgery, Stanford University School of Medicine, Stanford, CA, USA; 12 Department of Pathology, Johns Hopkins University School of Medicine, Baltimore, MD, USA

## Abstract

The role of glioma-associated myeloid cells in tumor growth and immune evasion remains poorly understood. We performed single-cell RNA sequencing of immune and tumor cells from 33 gliomas, identifying two distinct myeloid-derived suppressor cell (MDSC) populations in isocitrate dehydrogenase–wild-type (IDT-WT) glioblastoma: an early progenitor MDSC (E-MDSC) population with up-regulation of metabolic and hypoxia pathways and a monocytic MDSC (M-MDSC) population. Spatial transcriptomics demonstrated that E-MDSCs geographically colocalize with metabolic stem-like tumor cells in the pseudopalisading region. Ligand-receptor analysis revealed cross-talk between these cells, where glioma stem-like cells produce chemokines attracting E-MDSCs, which in turn produce growth factors for the tumor cells. This interaction is absent in IDH-mutant gliomas, associated with hypermethylation and repressed gene expression of MDSC-attracting chemokines. Our study elucidates specific MDSCs that may facilitate glioblastoma progression and mediate tumor immunosuppression.

## Graphical Abstract

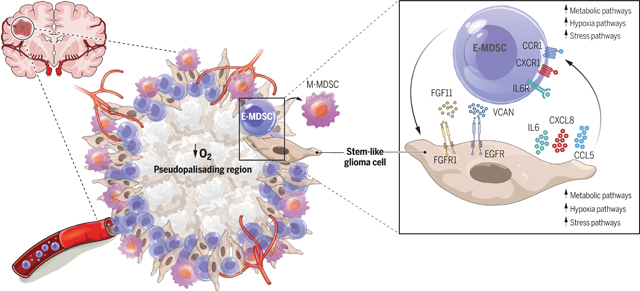

Checkpoint blockade immunotherapy targeting the T cell checkpoints CTLA-4, PD-1/L1, and recently LAG3 (1–3) has shifted the treatment standard of more than 18 solid tumors. However, the benefit of these immune-based therapies in gliomas has thus far been disappointing. Isocitrate dehydrogenase–wild-type (IDH-WT) glioblastoma, the most lethal adult-type diffuse glioma, is a quintessential example of an immunologically “cold tumor” with relatively few tumor-infiltrating lymphocytes, which greatly limits the effectiveness of T cell–targeted therapies. Nonetheless, true neoantigen-specific T cells have been demonstrated in glioblastoma (4). Taken together, these findings suggest that other cells and signals in the glioblastoma tumor microenvironment (TME) could suppress the function of tumor-specific T cells. Tumor-infiltrating myeloid cells are an important component of the TME, constituting >70% of tumor-infiltrating immune cells in glioblastoma (5). However, the precise composition and phenotypes of glioma-associated myeloid cells (GAMs), their role in shaping and interacting with other components of the glioblastoma TME, and their suitability as targets for therapeutic intervention are poorly understood.

To better characterize the cellular components of primary brain cancer, we performed single-cell RNA sequencing (scRNA-seq) on a cohort of gliomas (*n* = 33) spanning low to high grade, focusing on populations specifically acquired in IDH-WT glioblastomas. Surveying the transcriptomes of >750,000 immune cells and >350,000 tumor and associated stromal cells in these samples, we found a diverse landscape of myeloid-lineage cells in gliomas. Two immature bone marrow–derived myeloid cell (BMDM) populations, characteristic of recently described early progenitor and monocytic myeloid-derived suppressor cell (E-MDSC and M-MDSC) populations, are almost exclusively present in aggressive IDH-WT glioblastoma (6). Both populations of MDSCs demonstrated strong up-regulation of multiple catabolic and anabolic pathways with associated induction of hypoxia and stress response programs, with strong parallels to a subset of tumor cells. The E-MDSC population exclusively colocalizes with a tumor cell population with stem-like programs in the pseudopalisading region, a common histologic feature of glioblastomas. Receptor-ligand interaction mapping demonstrates cross-talk between these MDSCs and tumor cells, whereby the tumor cells produce multiple chemokines that could attract the MDSCs, and reciprocally, the MDSCs produce factors that can potentially promote the growth of the colocalized cancer cells. IDH-mutant gliomas (both IDH-mutant astrocytoma and IDH-mutant and 1p/19q-codeleted oligodendroglioma) showed promoter hypermethylation and silencing of these chemokine genes and do not harbor these MDSC populations. This large-scale single-cell atlas of glioma tumor and immune cells has thus revealed a potential critical interplay between glioma cells and MDSC populations in the pseudopalisading zone of IDH-WT glioblastomas.

## Diverse and distinct landscape of myeloid cells in glioma

**Fig. 1.**
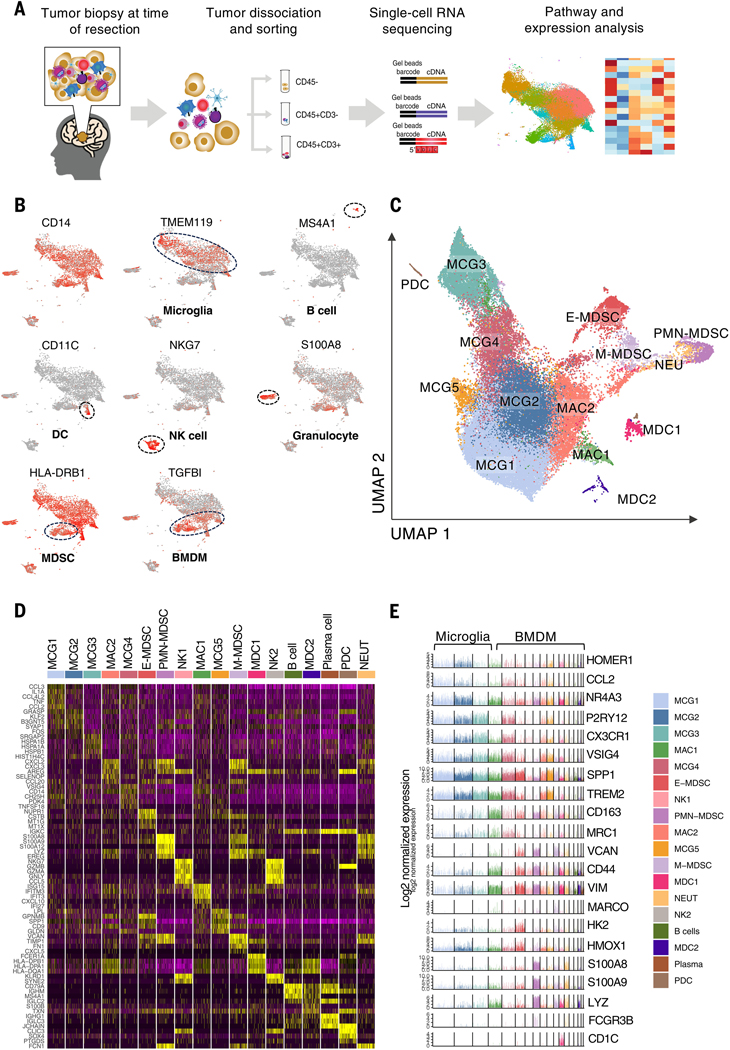
Profiling GAMs by scRNA-seq reveals diverse landscape. (**A**) Schematic graph showing the experimental design of scRNA-seq analysis of tumor and immune cells in gliomas. scRNA-seq was performed on CD45− and CD45+CD3− cells, and scRNA-seq and TCR-seq were performed on T cells isolated from resected IDH-WT glioblastoma *(n* = 21), IDH-mutant and 1p/19q-codeleted grade 2 oligodendroglioma (*n* = 6), IDH-mutant grade 2 astrocytoma (*n* = 1), IDH-mutant grade 3 astrocytoma (*n* = 3), IDH-mutant grade 4 astrocytoma (*n* = 2), and nonneoplastic cortex (*n* = 5). (**B**) UMAP projection of expression of canonical genes for microglia, BMDMs, granulocytes, B cells, natural killer (NK) cells, dendritic cells (DCs), and lack of expression of MHCII (HLA-DR) in MDSCs. (**C**) UMAP projection of the expression profile of myeloid-lineage cells in gliomas demonstrating 14 clusters of myeloid-lineage cells, including microglia (MCGs), macrophages (MACs), MDSCs, neutrophils (NEUs), monocyte-derived DCs (MDCs), and plasmacytoid DCs (PDCs), each delineated by color code. (**D**) *Z* score–normalized heatmap of top 5 differentially expressed genes with highest log fold change from each cluster. (**E**) Bar plots showing gene expression levels of select representative genes across microglia and BMDM clusters.

To characterize the diverse landscape of GAMs in glioma and healthy control patients, we performed scRNA-seq on freshly isolated fluorescence-activated cell sorting (FACS)– purified CD45+CD3−immune cells from 21 resected IDH-WT glioblastomas, 6 IDH-mutant and 1p/19q-codeleted grade 2 oligodendrogliomas, 1 IDH-mutant grade 2 astrocytoma, 3 IDH-mutant grade 3 astrocytomas, 2 IDH-mutant grade 4 astrocytomas, and 5 nonneoplastic brain tissue samples (Fig. 1A and table S1) (7). In total 240,183 CD45+CD3− immune cells passed quality control and were carried forward for analysis (fig. S1, A and B). Unbiased clustering and batch effect correction algorithms applied to the total combined transcriptomes of CD45+CD3− cells from all the analyzed tumors and nonneoplastic brain tissue samples identified 18 clusters of immune cells (fig. S2A). Canonical marker genes were used to identify major cell populations; myeloid cells with low expression of HLA-DR are characteristic of MDSCs (Fig. 1B). Focusing specifically on myeloid-lineage cells, we identified 14 clusters of myeloid cells, including 5 clusters of microglia and 9 clusters of BMDMs (Fig. 1C). Using differentially expressed genes by each cluster compared with other myeloid clusters, we annotated the individual clusters into distinct cell types (Fig. 1, D and E; fig. S2B; and table S2). Our single-cell profiling demonstrates a diverse and complex composition of microglial and BMDM populations across nonneoplastic and tumor-bearing CNS compartments that substantially expands upon the traditional view of M1 and M2 tumor-associated macrophages in gliomas.

## Distinct MDSC populations specifically present in IDH-WT glioblastomas

**Fig. 2.**
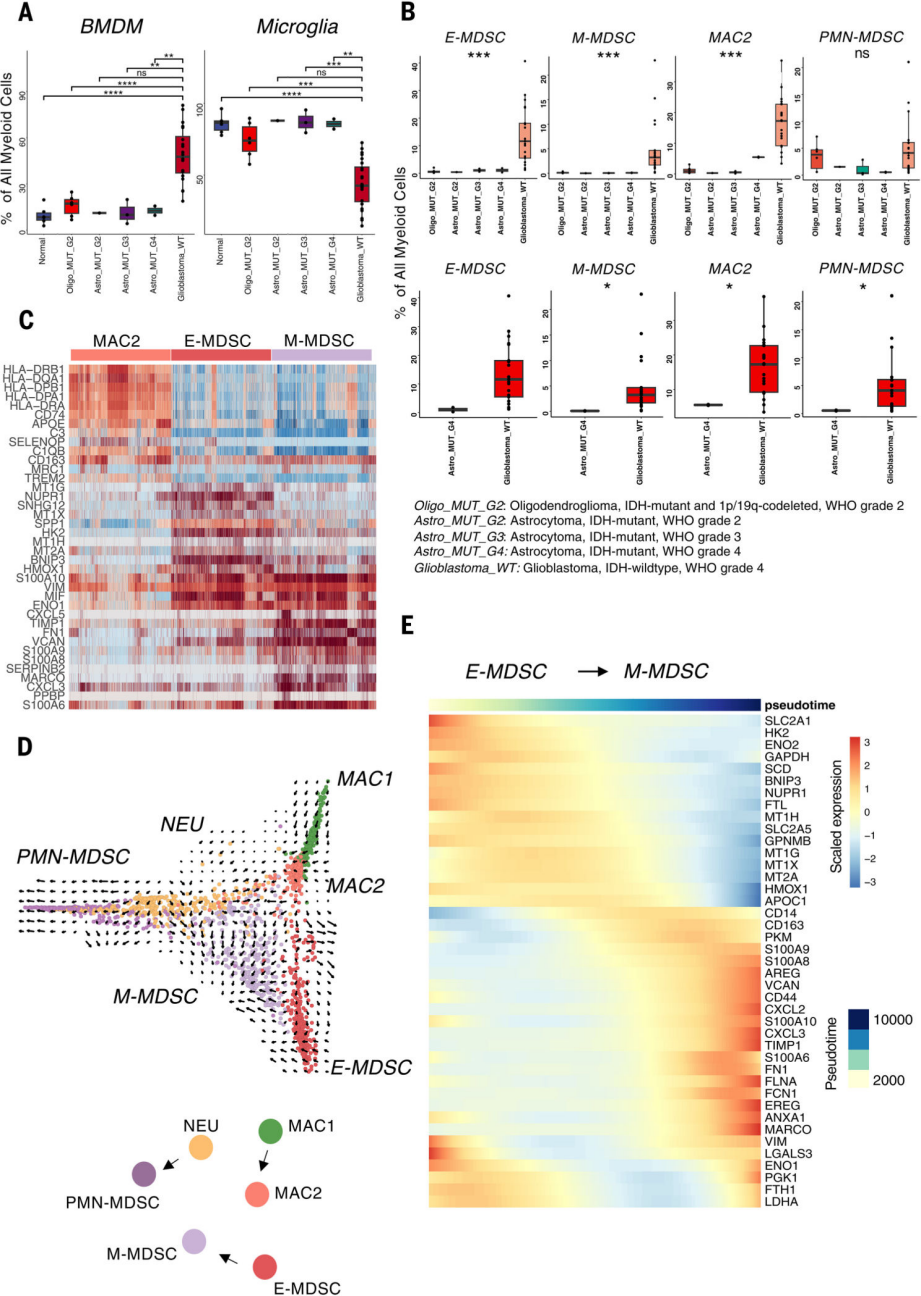
IDH-WT glioblastomas harbor specific MDSC populations that exist along a continuum of cellular states. (**A**) The proportion (percentage) of total myeloid-lineage cells made up by BMDMs and microglia compared between tumor grades and nonneoplastic tissue demonstrate increasing proportion of BMDMs and decreasing proportion of microglia with increasing tumor aggressiveness. Pairwise comparison and *P* values were obtained using Wilcoxon test. Each dot represents a patient, and all data points are shown. Individual data points are superimposed over a box and whiskers plot summarizing the data. *P ≤ 0.05; **P ≤ 0.01; ***P ≤ 0.001. (**B**) Box plots showing increased proportion of E-MDSC, M-MDSC, and MAC2 cell populations in IDH-WT glioblastoma compared with IDH-mutant gliomas, including grade 4 IDH-mutant astrocytomas. Comparisons and *P* values were obtained using Kruskal-Wallis (top) and Wilcoxon (bottom) tests. (**C**) Heatmap of differentially expressed genes between cells belonging to the three BMDM subsets enriched in IDH-WT glioblastoma demonstrating distinct transcriptomic profiles, with each column representing a cell. (**D**) Diffusion plot with RNA velocity for BMDM clusters in IDH-WT glioblastoma suggesting developmental linkage among BMDMs with E-MDSCs having the potential to transition to M-MDSCs. Cells were randomly downsampled to 300 cells per each cluster for visualization. (**E**) Heatmap demonstrating the pattern of gene expression along the E-MDSC–to–M-MDSC trajectory with increase in expression of genes associated with immune inflammation and scavenger receptors and decrease in expression of genes associated with metabolic pathways.

On the basis of evidence from murine models that specific myeloid populations in the TME can affect the growth and phenotype of tumor cells as well as antitumor immunity, we first investigated whether there were differences in the distribution of GAM populations as a function of tumor grade and molecular status. Although the distribution of GAMs varied across individual patients (fig. S2C), we found a large preponderance of BMDMs over microglia in more-aggressive tumor states, with increasing proportions of BMDMs correlating with increasing glioma grade (Fig. 2A). Notably, we found that only 2 of the 14 myeloid populations were specific to IDH-WT glioblastomas while being virtually absent in IDH-mutant grade 2 and 3 gliomas (Fig. 2B). One was a population resembling early-stage MDSCs (E-MDSCs) that lacked expression of lineage markers, and the other was a population of monocytic MDSCs (M-MDSCs) with stronger expression of CD14. A third myeloid population, designated MAC2, with transcriptomic features of both M1 and M2 macrophages, was also highly enriched in IDH-WT glioblastoma, although small numbers were observed in IDH-mutant lower-grade tumors. Notably, when comparing the presence of these MDSCs across tumors with different molecular features, these cells are exclusively present in IDH-WT glioblastomas compared with IDH-mutant grade 4 astrocytomas. There was no significant difference in the presence of these cells across other molecular features [i.e., epidermal growth factor receptor (EGFR) amplification and mutation status or O^6^-methylguanine-DNA methyltransferase (MGMT) methylation status] (fig. S2E).

Transcriptomic analysis of these three populations revealed that E-MDSCs preferentially expressed multiple genes encoding metabolic enzymes, stress-induced genes, and metallothionines; M-MDSCs exhibited preferential expression of multiple S100A family genes and cellular migration genes; and the MAC2 macrophage population expressed high levels of MHC2, scavenger receptors, and tissue damage–associated genes (Fig. 2C). No other myeloid population was selectively present in IDH-WT glioblastomas. Although the role of MDSCs in the inhibition of natural antitumor T cell responses in T cell–poor tumors is unclear, nonetheless, a standard test of MDSC function is inhibition of T cell proliferative responses in vitro. The glioblastoma-infiltrating MDSCs were separated by FACS into M-MDSC (CD14+), PMN-MDSC (CD15+), and E-MDSC (CD14−, CD15−, and CD16−) populations (fig. S3A and table S3). Cell trace violet (CTV)–labeled, anti-CD3/CD28− stimulated peripheral blood mononuclear cells (PBMCs) were cocultured independently with each tumor-associated MDSC subset at varying MDSC:PBMC ratios. We found that all three MDSC subsets robustly suppress T cell proliferation (assayed by CTV dilution), with M-MDSCs demonstrating the strongest in vitro suppression of both CD4 and CD8 T cell proliferation (fig. S3, B to D). These results validate that the MDSCs defined transcriptionally in our single-cell analysis exhibit a classic functional feature of MDSCs.

## E-MDSC, M-MDSC, and GAM exist on a continuum of cellular states

To infer the differentiation trajectory and transcriptional states of the GAMs, we generated diffusion maps and performed pseudotime and RNA velocity analysis on the BMDM cells, specifically focusing on the MDSC and macrophage populations. We found developmental linkage among three pairs of glioblastoma-specific myeloid populations (E-MDSC→M-MDSC, MAC1→MAC2, PMN→PMN-MDSC). E-MDSCs are the earliest in the developmental trajectory and have the potential to develop along a distinct developmental trajectory to M-MDSCs (Fig. 2D).

Next, to identify specific genes that are dynamically changing along the single-cell trajectory from E-MDSCs to M-MDSCs, we used pseudotime analysis to evaluate global transcriptomics along individual differentiation trajectories vectorially established through our RNA velocity analysis (Fig. 2E). We found that as cells transition between E-MDSC and M-MDSC states, there is increased expression of genes associated with extracellular matrix components and remodeling *(CD44, FLNA, VCAN,* and *FN1),* immune inflammation *(FCN1* and *S100* proteins), chemokines *(CXCL2* and *CXCL3),* and monocytic scavenger receptors *(CD14, MARCO,* and *CD163)* generally associated with M2 macrophages. Conversely, there is a decrease in expression of genes associated with metabolic pathways, including glycolysis *(HK2, ENO2,* and *SCD),* antioxidant pathways *(HMOX1, MT1G,* and *MT1H),* and cellular stress response *(BNIP3* and *NUPR1)* (Fig. 2E and fig. S4). The down-regulation of metabolic and hypoxia pathways in the setting of cellular transition from the E-MDSC to M-MDSC states suggests differences in the microenvironmental milieu of the TME that these cells occupy.

## IDH-WT glioblastoma MDSCs exhibit robust catabolic and anabolic metabolism

**Fig. 3.**
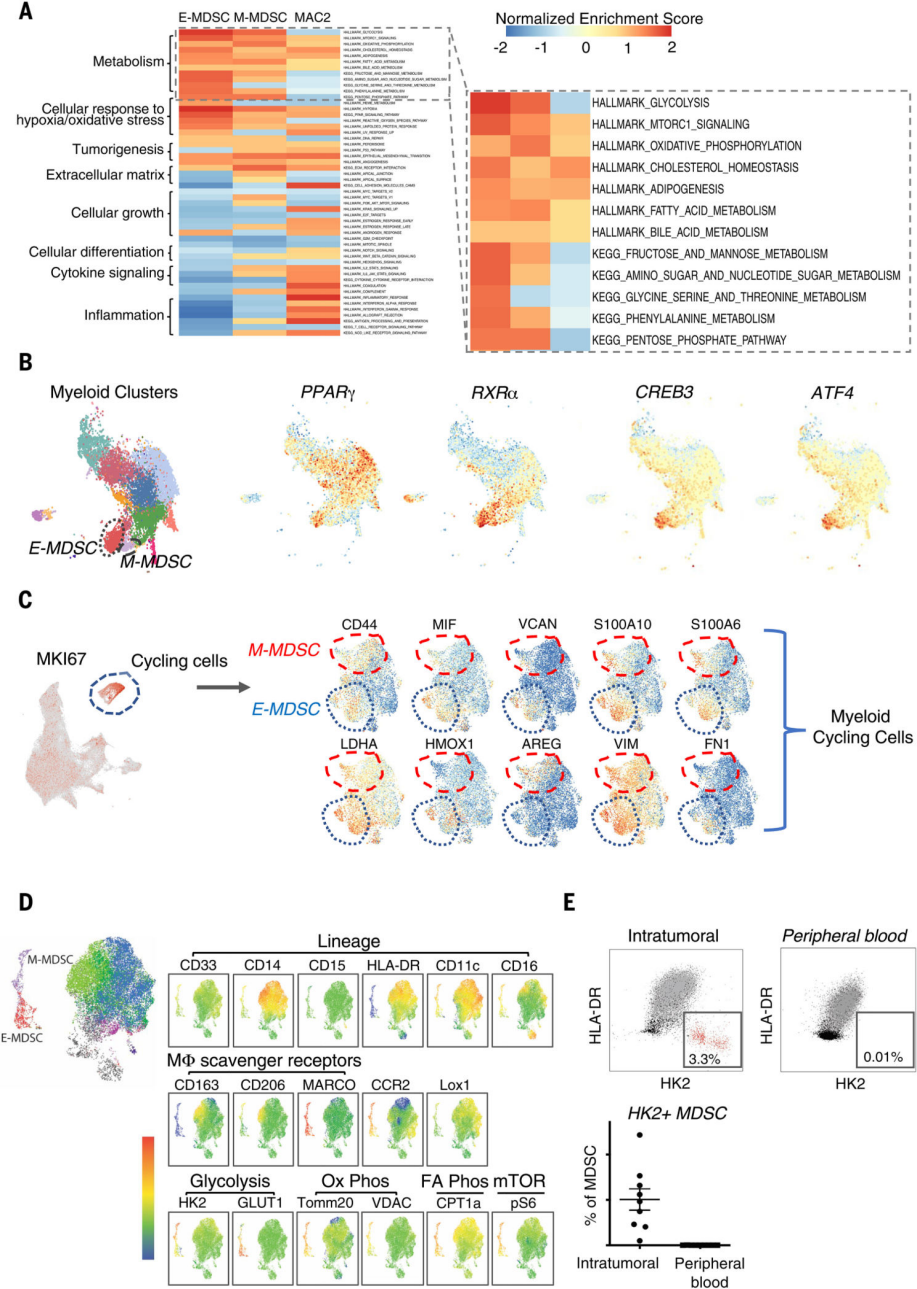
Glioblastoma-specific MDSCs exhibit distinct metabolic pathways. (**A**) Heatmap of Hallmark and KEGG GSEA enriched pathways among E-MDSC, M-MDSC, and MAC2 cell populations. Enlarged panel demonstrates the top up-regulated metabolic pathways in MDSCs. (**B**) UMAP plots demonstrating increased activation of transcription factors that regulate carbohydrate, fatty acid, and amino acid metabolism in MDSCs. (**C**) UMAP plot demonstrating population of myeloid cells with high expression of cycling gene (MKI67) (left). Subclustering of cycling myeloid cells demonstrates that E-MDSCs and M-MDSCs represent close to half of the cycling cells. UMAP plots of subclustered cycling cells demonstrate the large number of cells exhibiting high expression of genes specifically found in E-MDSC and M-MDSC cells (right). (**D**) t-distributed stochastic neighbor embedding (t-SNE) plots of multicolor flow cytometry, illustrating the presence of two populations of myeloid cells with low expression of HLA-DR and high expression of proteins involved in glycolysis, oxidative phosphorylation, and mTOR metabolic pathways recapitulating E-MDSC and M-MDSC populations identified through scRNA-seq (MΦ, macrophage). (**E**) Flow cytometry depicting the distinctive presence of HK2+ MDSCs with high expression of signature proteins in various metabolic pathways in the TME compared with PBMCs.

Given the substantial shifts in pseudotemporal expression of genes associated with metabolic pathways as cells transition between different myeloid cellular states, we performed gene set enrichment analysis (GSEA) to further characterize pathways representative of functional states of the MDSC populations. GSEA analysis of differentially expressed genes in E-MDSCs and M-MDSCs compared with other BMDM subsets revealed that the most prominently induced gene sets were dominated by multiple pathways of catabolic and anabolic metabolism, including enrichment of glycolysis, oxidative phosphorylation, fatty acid metabolism, and mammalian target of rapamycin (mTOR) pathways (Fig. 3A). The most enriched gene set pathways found in both populations of MDSCs were carbohydrate and lipid metabolism pathways. In addition to glycolysis and fatty acid metabolism, E-MDSCs also exhibited up-regulation of metabolism of other carbohydrates, including fructose and mannose, the pentose phosphate pathway, nucleotide and amino sugar metabolism, as well as amino acid metabolism. Using SCENIC (8), a program that determines the activation state of specific transcription factors based on expression levels of their regulon, we identified E-MDSC–specific activation of transcription factors that regulate metabolic pathways, including the *CREB, ATF, PPAR,* and *RXR* families of transcription factors critical to homeostasis of glucose, lipid, and amino acid metabolism (Fig. 3B). Examining programs more closely in E-MDSCs versus M-MDSCs, E-MDSCs appeared to have higher activation of these transcription factors (Fig. 3B) and demonstrated a significant increase in the expression of genes encoding key enzymes involved in glycolysis: hexokinase-2 *(HK2)* and glyceraldehyde 3-phosphate dehydrogenase *(GAPDH).* This population of cells also showed increased expression of glucose transporter 1 *(SLC2A1)* that allows the influx of glucose into the cell as a substrate for glycolysis (fig. S5, A and B). These broad anabolic and catabolic transcriptional programs are highly indicative of an active proliferating population. In fact, E-MDSCs and M-MDSCs represented the major components of a proliferating myeloid cluster defined by genes such as *MKI67.* Subclustering of cycling myeloid cells demonstrate that E-MDSCs and M-MDSCs represent close to half of the cycling cells (Fig. 3C).

To validate the major metabolic programs of glioblastoma-associated MDSCs at the protein level, we performed 21-color multiparametric flow cytometry on 10 additional IDH-WT glioblastoma tumor samples (table S4). Using a combination of antibodies to molecules associated with various myeloid lineages and metabolic enzymes and markers (9), we identified two distinct populations of HLA-DR−CD33+ cells that recapitulated the MDSC subsets identified from the scRNA-seq analysis (Fig. 3D). In particular, we could discern an MDSC population that recapitulated high expression of HK2 and GLUT1 similar to the E-MDSC population identified through scRNA-seq. We also identified a separate population of MDSCs with higher expression of CD14 and CD206 and lower expression of GLUT1, similar to the M-MDSC population identified through scRNA-seq. Although both MDSC populations demonstrated up-regulation of glucose metabolism, E-MDSCs exhibited higher expression of GLUT1 compared with M-MDSCs. We hypothesized that M-MDSCs may use a different glucose transporter. Gene expression analysis found that they displayed higher expression of GLUT3, whereas E-MDSCs displayed higher expression of GLUT1 and GLUT5 (fig. S5C). Our flow cytometry results also demonstrated increased expression of voltage-dependent anion-selective channel 1 (VDAC1), mitochondrial import receptor subunit TOM20 homolog (TOMM20), and phosphorylated S6 ribosomal protein (pS6) in these cells, which are a part of the oxidative phosphorylation and mTOR pathways, respectively, compared with HLA-DR+ macrophages. Taken together, these proteomic studies validated our single-cell transcriptomic profiling, demonstrating significant up-regulation of multiple metabolic pathways in the MDSCs (fig. S5D). We further performed flow cytometric analysis on matched PBMCs from the same glioblastoma patients whose tumors recapitulated protein validation of the scRNA-seq analysis. These analyses showed that HK2+ MDSCs were exclusively found among tumor-infiltrating myeloid cells and not in the peripheral blood, further supporting our hypothesis that these cells alter their metabolic pathways in response to the nutrient-poor TME (Fig. 3E). Taken together, these findings demonstrate that glioblastoma MDSCs are highly programmed to take up and metabolize nutrients that are limited in the TME for high-energy needs as well as rapid cellular expansion via anabolic pathways. These up-regulated metabolic programs are characteristic of tumor cells themselves but have not been previously recognized in MDSCs in the TME.

## MDSCs demonstrate selective up-regulation of hypoxia and cellular stress responses

We hypothesized that these cells exhibit highly metabolic genetic programs because of the nutrient-poor TME and, therefore, would also exhibit up-regulation of other functional programs that allow them to persist. Our results further demonstrated that both populations of MDSCs displayed up-regulation of genes and transcription factors associated with cellular stress and oxidative stress response, including hypoxia, reactive oxygen species, and heme metabolism pathways, all of which have previously been implicated to play important roles in adaptive and innate immune dysfunction and tumorigenesis (10) (fig. S6, A and B). One particular gene that demonstrated significant up-regulation in the E-MDSCs is *HMOX1,* the rate-limiting enzyme in the catabolism of free heme that plays a key role in regulating anti-inflammatory and antioxidant pathways.

Coincidentally, these cells also exhibited increased expression of ferritin light and heavy chains (*FTL* and *FTH1*) (fig. S5A). On transcription factor analysis, E-MDSCs demonstrated increased activation of transcription factors that regulate the expression of *HMOX1*. In addition to *HMOX1*, E-MDSCs also exhibited increased expression of genes (*NUPR1* and *ERO1A*) and associated transcription factors linked to cellular response to hypoxia (fig. S5B). M-MDSCs also up-regulated gene pathways involved in hypoxia and stress response as well as genes encoding growth factors (*EREG*), chemokines (*CXCL2* and *CXCL3*), and extracellular matrix (ECM) components that are key facilitators of cell motility. Our results reveal that these distinct populations of E-MDSC and M-MDSC cells specifically present in IDH-WT glioblastomas not only up-regulate their metabolic programming to competitively survive in a nutrient-poor microenvironment but also up-regulate pathways associated with cellular response to a low-oxygen environment, including antioxidant and cellular repair pathways to acclimate, thrive, and even actively proliferate (Fig. 3C) in the hostile milieu of IDH-WT glioblastoma.

## Potential ligand-receptor interactions and spatial colocalization between E-MDSCs and glioma cells with stem-like and mesenchymal programs

**Fig. 4.**
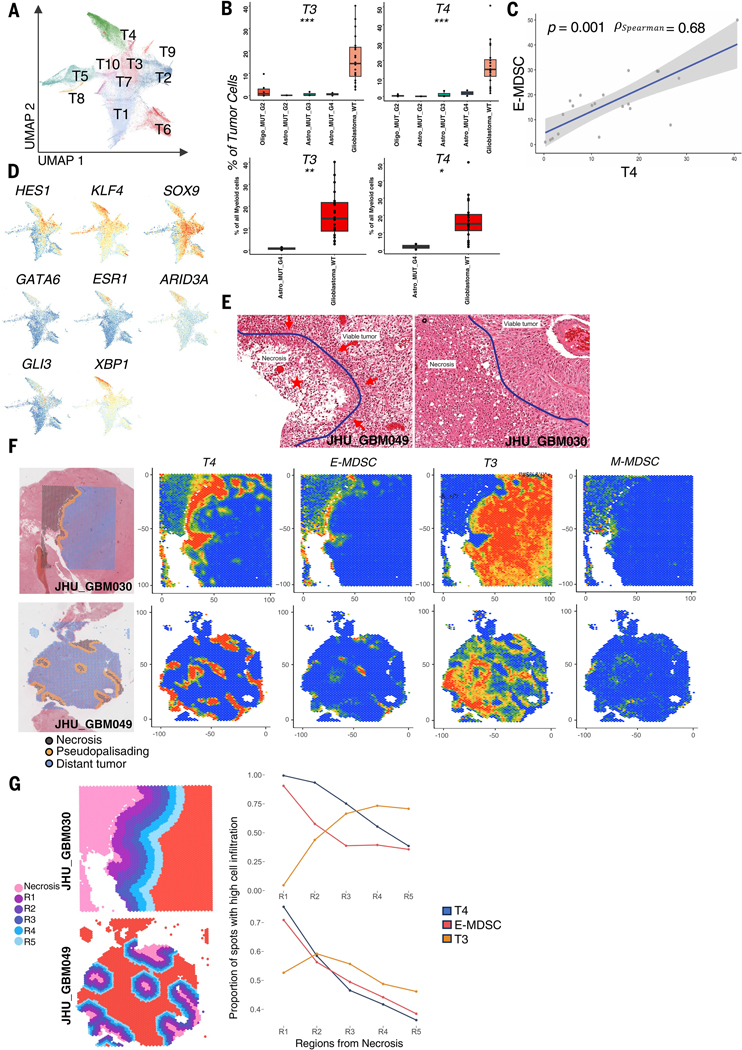
E-MDSCs colocalize with glioblastoma-specific tumor cells with mesenchymal and stem-like programs to the pseudopalisading region of tumor. (**A**) UMAP projection of the expression profile of neoplastic cells demonstrating 10 clusters of tumor cells, each delineated by color code. (**B**) Box plots showing significant overrepresentation of tumor clusters T3 and T4 in IDH-WT glioblastoma compared with grade 2 IDH-mutant and 1p/19q-codeleted oligodendrogliomas and grades 2, 3, and 4 IDH-mutant astrocytomas. *P ≤ 0.05; **P ≤ 0.01; Kruskal-Wallis and Wilcoxon tests. (**C**) Scatter plot highlighting the Spearman correlation between the frequencies of E-MDSC and T4 across IDH-WT glioblastoma patients. (**D**) UMAP plots demonstrating activation scores of transcription factors that promote cancer stemness activated preferentially in glioma stem-like tumor cluster T4. (**E**) Representative H&E pathological slides of IDH-WT glioblastoma samples demonstrating the pseudopalisading region characterized by hypercellular cells (arrows) surrounding a central area of necrosis (red star). (**F**) Labeling at the spot level of the necrotic (gray), pseudopalisading (yellow), and rest of the distant tumor (blue) regions of two representative patients overlayed on H&E pathological slides (left). Deconvolution of Visium spatial transcriptomics demonstrating high probability of the presence of E-MDSC, M-MDSC, and T4 populations at the pseudopalisading region of glioblastoma. It also demonstrated the presence of T3 localized directly adjacent to T4 (right). (**G**) Distance from necrosis was divided into five regions (R1 to R5), and the proportion of E-MDSC, T4, and T3 populations within each spot is plotted as a function of distance from necrosis, which demonstrates decreased proportion of E-MDSC and T4 and increased proportion of T3 cells as we move farther away from necrosis and the pseudopalisading region.

To define the potential interplay between the myeloid cells and tumor cells, we next carried out unsupervised clustering analysis of the CD45− cell population (fig. S7A), classifying cells into malignant and nonmalignant cell types by inferring copy number variations (CNVs) on the basis of the expression intensity of genes across positions of the genome (inferCNV) (11) (fig. S7B). Ten clusters of tumor cells were defined in our uniform manifold approximation and projection (UMAP) analysis with meta-programs consistent with previous studies (12) (Fig. 4A). We next evaluated the presence of tumor cell populations across glioma subtypes and found that tumor populations T3 and T4 are highly selectively expressed in IDH-WT glioblastomas, whereas they are virtually absent in IDH-mutant gliomas. Similar to what we observed with the MDSCs, these particular populations of tumor cells are also enriched in IDH-WT glioblastomas (Fig. 4B and fig. S7C).

To determine potential interactions between myeloid and tumor cells in the glioblastoma TME, we performed pairwise Spearman correlation analysis on the proportion of the various transcriptionally determined subpopulations of tumor and immune cells across our patient samples. Notably, frequencies of E-MDSC cells showed significant positive correlation with the presence of only one of the tumor clusters, T4, across all samples and within IDH-WT glioblastomas (Fig. 4C). Transcriptomic analysis of the T4 tumor populations revealed increased expression of genes associated with angiogenesis (*VEGFA*), neuronal development (*MALAT1*), hypoxia response (*ERO1A*), and glycolysis (*PGK1*). Correspondingly, on GSEA analysis, the T4 tumor cluster demonstrated up-regulation of tumorigenesis pathways, including VEGF signaling, integrin, glycolysis, and hypoxia pathways (fig. S7, D and E). These pathways have previously been demonstrated to be up-regulated and drive stemness in glioma cells (13, 14).

Regulon-based transcription factor activity analysis demonstrated that this T4 tumor cell population up-regulated activity of transcription factors previously described in cells that demonstrate stem-like features that play key roles in reprogramming differentiated IDH-WT glioblastoma cells into stem-like cells capable of self-renewal and tumor propagation, including HES1, SOX9, and KLF4 (15). The T4 population also exhibited activation of ARID3A, GLI3, GATA6, and XBP1 programs, which are less described in gliomas but have been implicated in inducing stem-promoting pathways, such as Hedgehog signaling pathway, and maintaining the stemness of cancer cells in other systemic cancers (16–21) (Fig. 4D). Although it is unclear whether glioma stem cells (GSCs) are the one renewable tumor cell subset giving rise unidirectionally to other tumor subsets, they are a key subset of glioma tumor cells that are responsible for tumor progression, invasion, and resistance to chemoradiation (22). With the advancement in techniques to study human gliomas, there has been evolution in the GSC models that traditionally have been discussed predominantly in mouse models and in vitro systems. Recent scRNA-seq efforts in human gliomas have provided additional granularity to cell states in glioblastoma, with multiple cell states capable of propagating tumor growth (12). Transcriptomic and transcription factor programs in our T4 cell population are reminiscent of proliferating and hypoxic mesenchymal cellular states previously described. These cell states have been shown to have progenitor phenotypes and tumor propagation potential (23, 24). Our RNA velocity analysis of the tumor cell clusters suggests that tumor cluster T4 has the potential to transition to cluster T3, which has the potential to transition into other tumor clusters, indicating a stem-like role of the T4 cluster (fig. S7F).

Given the shared up-regulation of metabolic pathways associated with high energy demands in low-oxygen and nutrient-limited environments—such as glycolysis, angiogenesis, and hypoxia pathways—between the MDSC and T4 tumor cell populations, we hypothesized that these cells might geographically colocalize in tumor regions, requiring enhanced activity of these pathways to support cellular growth and energy consumption under nutrient and oxygen constraints. To further evaluate the potential colocalization of these populations, we profiled spatial gene expression from two representative IDH-WT glioblastoma patients from our cohort across different histological regions of the tumor, including necrosis, pseudopalisading regions, and distant tumor areas. Each sample consisted of 5-μm tissue sections analyzed by the Visium spatial transcriptomics platform. Dimensionality reduction and clustering of spatial transcriptomic features identified clusters with differential gene expression resembling clusters E-MDSC and T4 cell populations identified through scRNA-seq analysis. These were specifically localized to the pseudopalisading region of the tumor (fig. S8, A and B). The pseudopalisading region of glioblastoma is a hypercellular zone surrounding a necrotic focus (Fig. 4E) that is a distinguishing pathological feature of IDH-WT glioblastoma. This region is characterized by a hypoxic microenvironment and microvascular proliferation. The presence of pseudopalisading necrosis and microvascular hyperplasia are significant predictors of poor prognosis in gliomas, and the hypoxic environment is thought to drive tumor cell survival and stemness (25). Although these regions have traditionally been thought to be predominately made up of tumor cells, our results suggest that there is a substantial contribution to the cellular architecture of the pseudopalisading region by the MDSCs. Using robust cell type decomposition (RCTD), we profiled the spatial localization of E-MDSC and T4 defined in our scRNA-seq data and found a virtually complete colocalization of E-MDSC and T4 to the pseudopalisading region of IDH-WT glioblastoma with high probability and, to a lesser extent, M-MDSCs, which are adjacent to rather than colocalized with the T4 tumor subset (Fig. 4F) (26). To further quantify the localization of E-MDSC and T4 to the pseudopalisading region to the other cell populations, we divided the regions on the basis of distance from necrosis into five regions and calculated the probability of the presence of specific clusters of cells in each region. We found that the probability of the presence of E-MDSC and T4 were highest in region 1 (closest to the necrotic zone), and as we move farther out from the necrotic zone, the probability decreases accordingly. By contrast, for other cell populations, such as T3, the probability increases as we move further out from necrosis (Fig. 4G).

## Cluster interactions suggest a symbiotic relationship between E-MDSCs and mesenchymal glioma stem-like cells

**Fig. 5.**
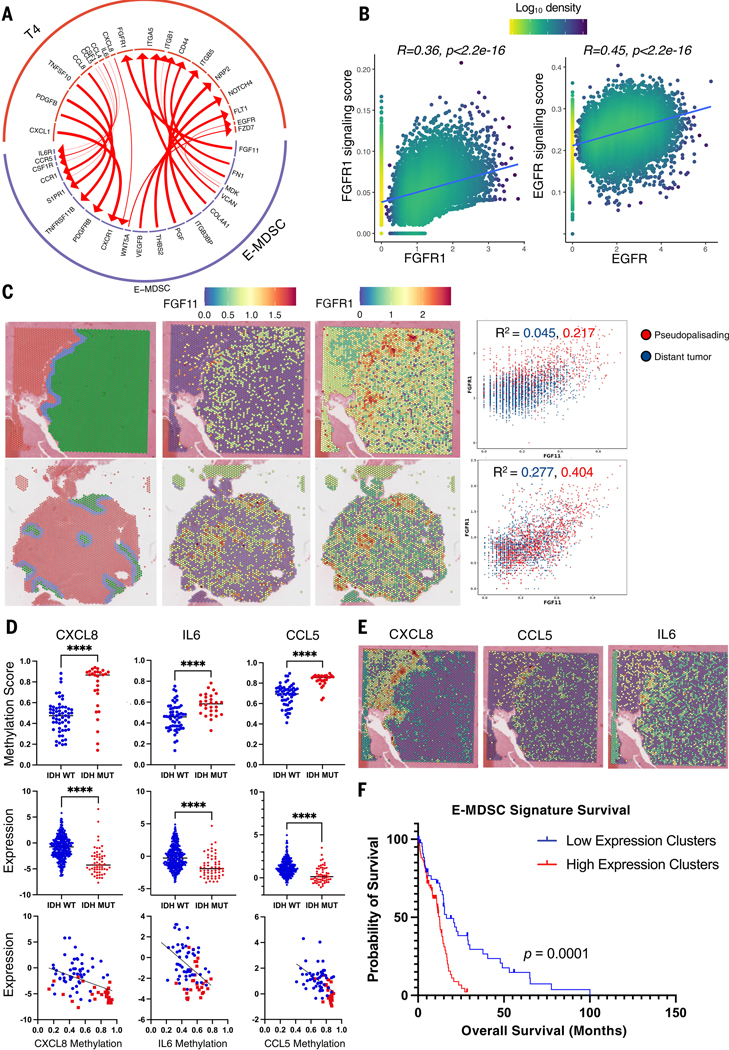
E-MDSC and T4 stem-like cells demonstrate a potential symbiotic relationship highlighted by MDSC recruitment and tumor promotion. (**A**) Predicted ligand-receptor interactions between E-MDSC and tumor cell T4 populations in IDH-WT glioblastoma. (**B**) Correlation analysis of growth factor expression with gene set signaling score in T4 tumor cells demonstrating strong correlation between the expression level of E-MDSC stimulated growth factor receptor to downstream signaling pathway. (**C**) Spot-level plot of normalized expression of ligand-receptor pair FGF11 and FGFR1 enriched in the pseudopalisading region (left); correlation of adjacent expression of FGF11 and FGFR1 demonstrated increased coexpression within the pseudopalisading region compared with distant tumor (right). (**D**) Dot plots and correlation plots depicting decreased methylation score and increased expression of MDSC recruiting chemokines in IDH-WT glioblastomas. (**E**) Normalized expression of key MDSC recruiting and activating chemokines and cytokines demonstrated localized expression to the pseudopalisading region. (**F**) Kaplan-Meier curve illustrating that the E-MDSC expression signature stratified IDH-WT glioblastoma patient survival.

The colocalization of E-MDSCs with glioma cells that have stem-like mesenchymal features in the pseudopalisading region of IDH-WT glioblastomas suggests that short-range communications between these cells can potentially enhance tumor aggressiveness and progression, including signals from membrane ligands, cytokines, chemokines, and growth and differentiation factors. We therefore examined cognate ligand-receptor interactions selectively increased between E-MDSC and T4 tumor cells to determine putative modes of communication between these cells. We used an established dataset of biologically validated ligand-receptor pairs to score interactions between pairs of cell clusters (27, 28) (table S5). We found markedly high numbers of ligand-receptor pair interactions between E-MDSC and T4 tumor cells (Fig. 5A).

Multiple predicted receptor-ligand interactions suggested an active role for the tumor cells (T4) in the recruitment and proliferation of E-MDSCs. Our results demonstrated increased chemokine-chemokine receptor signaling from T4 to E-MDSCs through several ligand-receptor pairs: *CCL8-CCR1*, *CXCL1-CXCR1*, *CCL5-CCR1*, *CXCL8-CXCR1*, and *CCL4-CCR5*. Some of these interactions have been shown to participate in the recruitment of MDSCs into sites of inflammation and the TME in various murine cancer models, including gliomas (29–31). In addition to these chemokines, interleukin-6 (IL-6)– interleukin-6R (IL-6R) interactions further enhance MDSC accumulation and the induction of genes encoding various immune inhibitory functions in these cells (32). Tumor cluster T4 also demonstrated increased expression of macrophage colony-stimulating factor 1 (*CSF1*), a major myeloid growth factor, paired with high expression of *CSF1R* on E-MDSCs.

Reciprocally, we identified strong ligand-receptor interactions from E-MDSC to tumor cells with mesenchymal and stem-like programs driving tumor proliferation, survival, and invasion. Extracellular matrix component versican (*VCAN*) is strongly induced on E-MDSCs, whereas its corresponding receptor CD44 is induced in T4. E-MDSCs also demonstrated up-regulation of ligands for multiple growth factor receptor kinases. In particular, we identified a previously unknown *FGF11-FGFR1* interaction, which can promote tumor cell proliferation and survival through activation of Ras/MAPK, PI3K/AKT, and the phospholipase (PLC)/protein kinase C (PKC) signaling cascade (33, 34). Although FGFR1 has been reported as a potential IDH-WT glioblastoma growth factor receptor, our data suggest that E-MDSC–produced FGF11 may be its major ligand in glioblastoma. Furthermore, E-MDSC demonstrated increased inferred interaction with receptors on T4 tumor cells that are associated with tumor invasion, angiogenesis, and glioma cell migration, including integrin subunit alpha 5 (*FN1-ITGA5*) and vascular endothelial growth factor B (*VEGFB-FLT1*) (Fig. 5A). Transcription factor analysis of the tumor cells demonstrated increased activation of transcription factors associated with growth factor and angiogenesis pathways, including c-Jun, c-Myc, and ATF in the T4 subset (fig. S9A).

To further support our hypothesis that growth factors produced by E-MDSCs (Fig. 5A) are potentially engaging growth factor receptors on T4 tumor cells with subsequent activation of protumorigenic gene programs, we next generated signaling pathway scores from established gene sets for the key growth factor receptors—EGFR and FGFR1—that demonstrated up-regulation in the T4 tumor cell population identified from the ligand-receptor interaction analysis (35–37) (table S6). These signaling scores were then correlated to the expression level of the respective growth factor receptors for each cell in the T4 population. We found strong correlations between the expression level of the growth factor receptor with the associated signaling score of the growth factor pathway in each cell (Fig. 5B). This supports the notion that the inferred ligand-receptor interactions in Fig. 5A result in significant up-regulation of growth factor pathways in T4 tumor cells.

We further evaluated some of the ligand-receptor interactions identified by single–cell cluster interaction mapping between E-MDSC and T4 using our spatial transcriptomic data and found coenrichment in gene expression of representative ligand-receptor pairs in the pseudopalisading region, especially FGF11 and FGFR1 (Fig. 5C). Mining The Cancer Genome Atlas (TCGA) datasets, we found that the E-MDSC gene signature demonstrated a strong correlation with the tumor T4 population gene signature (fig. S9B). Taken together, colocalization of E-MDSC and T4 in the pseudopalisading regions of IDH-WT glioblastoma, together with ligand-receptor interaction analysis, support a model of a symbiotic relationship between the E-MDSC and T4 cells, in which the tumor promotes E-MDSC accumulation and proliferation, and E-MDSC accumulation supports tumor growth and invasion.

## MDSC signature is an independent prognostic factor in IDH-WT glioblastoma, with its recruitment potentially through epigenetic regulation

Given the specific recruitment of MDSCs in IDH-WT glioblastomas compared with their counterpart IDH-mutant grade 4 astrocytomas, we hypothesized that IDH1 or IDH2 (IDH1/2) mutation status is mechanistically linked to MDSC recruitment. Mutant IDH1/2 is known to produce and lead to accumulation of the oncometabolite 2-hydroxyglutarate (2-HG), which can inhibit the activity of ten-eleven translocation (TET) enzymes that mediate the DNA demethylation pathway. Consequently, IDH-mutant gliomas have been associated with a hypermethylator phenotype. We hypothesized that IDH-mutant gliomas may harbor hypermethylation and silencing of the puttive cytokines and chemokines associated with activation and recruitment of MDSCs. To test this hypothesis, we analyzed transcriptomic and methylation data from combined low-grade and high-grade TCGA cohorts (TCGA 2016) and found that IDH-WT glioblastomas do in fact demonstrate decreased methylation scores and corresponding increased expression of chemokines and cytokines, such as CCL5, CXCL8, and IL-6, compared with their IDH-mutant counterparts (Fig. 5D). When we analyzed the methylation data limited to high-grade gliomas (TCGA 2013, based on prior classification at the time of the database), we found a trend toward increased methylation of these genes with IDH-mutant grade 4 astrocytomas compared with IDH-WT glioblastomas, although methylation data on IDH-mutant grade 4 astrocyoma samples was limited (fig. S10A). We further found enrichment in gene expression of some of these chemokine and cytokines to the pseudopalisading region on a spatial level (Fig. 5E). To determine whether the presence of these specific populations of MDSCs and T4 tumor cells conferred differences in survival among glioblastoma patients, we examined the clinical consequences of expression of MDSC and T4 tumor cell–associated genes in bulk TCGA gene expression. When analyzing the combined low-grade glioma and high-grade glioma TCGA cohort, high–E-MDSC– and –M-MDSC–expression samples were almost exclusively IDH-WT glioblastomas, whereas low-expression samples contained high fractions of IDH-mutant tumors (fig. S10B), compatible with our scRNA-seq results that these populations of cells are specific to IDH-WT glioblastomas. We further subsetted TCGA samples into just IDH-WT glioblastomas, and survival analysis within IDH-WT glioblastomas alone demonstrated that glioblastoma patients with high E-MDSC gene expression signatures had significantly shorter overall survival compared with patients with low E-MDSC gene expression signatures, independent of MGMT methylation status (Fig. 5F). Similarly, M-MDSC and T4 gene expression signatures also stratified IDH-WT glioblastoma patient survival (fig. S10B). This suggests that the presence of these cells is an independent prognostic indicator in IDH-WT glioblastomas. To determine the association between methylation and expression of critical chemokines and the presence of E-MDSC signature, we clustered the combined glioma cohort on the basis of expression of E-MDSC gene signature and found that patients with low E-MDSC expression had decreased expression and hypermethylation of the above chemokines (fig. S10C).

## Discussion

Our comprehensive scRNA-seq, spanning low- to high-grade gliomas, integrated with spatial transcriptomic analyses, reveals a previously unrecognized interaction between an MDSC population and a tumor population with stem-like and mesenchymal transcriptomic programs associated with IDH-WT glioblastoma. Our transcriptomic, proteomic, and functional profiling identified two highly proliferative populations of MDSCs (E-MDSCs and M-MDSCs) specifically present in IDH-WT glioblastomas compared with IDH-mutant gliomas, including IDH-mutant grade 4 astrocytomas. These MDSCs exhibited up-regulation of various catabolic and anabolic pathways and stress and hypoxia response pathways, indicating a metabolic reprogramming conducive to rapid expansion within the TME. Moreover, our findings revealed a dynamic continuum of cellular states among MDSCs with immature E-MDSCs capable of transitioning into M-MDSCs, which suggests a multifaceted role in tumor progression and immune evasion. Notably, E-MDSCs localized within the pseudopalisading regions of IDH-WT glioblastomas, colocalizing with a tumor subset bearing the transcriptional programs of previously described mesenchymal stem-like cells with tumor propagation potential. Transcriptomic analysis highlighted the potential reciprocal interaction between E-MDSCs and glioma cells with stem-like and mesenchymal programs, mediated through chemokine signaling by the stem-like tumor cells that recruit and activate E-MDSCs specific to IDH-WT glioblastomas through epigenetic regulation. The E-MDSCs reciprocally may facilitate stem-like tumor cell growth and mesenchymal differentiation through the secretion of growth and angiogenetic factors.

Among patients with gliomas, IDH-WT glioblastoma patients were found to have significant elevation of MDSCs in the periphery with increased levels of MDSCs being associated with worse prognosis (38, 39). However, the distinct transcriptomic profile of subsets of MDSCs in IDH-WT glioblastomas and the precise mechanism by which MDSCs traffic to the tumor to promote tumor growth and immunosuppression has never been previously ascertained. Previous studies have demonstrated diversity in the landscape of glioma myeloid cells in humans and murine models, highlighting differences between tumor grades, newly diagnosed versus recurrent tumors, and different regions of the tumor. They have also revealed that myeloid cells in IDH-WT glioblastomas can significantly influence the state and phenotypes of tumor cells and T cells in the microenvironment (40–43). However, despite these advances, the source and biological implication of specific myeloid populations preferentially recruited to glioblastomas were not well understood. Moreover, studies on interactions between myeloid cells, tumor cells, and other immune cells in the microenvironment have mainly relied on inferred signaling pathways without comprehensive spatial analyses to elucidate colocalization and prospective close-range communication among these cell populations.

We identified three populations of MDSCs in glioblastomas, including E-MDSCs, M-MDSCs, and PMN-MDSCs, with IDH-WT glioblastomas being significantly enriched in E-MDSCs and M-MDSCs. Both populations of cells, particularly E-MDSCs, exhibited a robust and diverse sets of metabolic genes with up-regulation of glycolysis, oxidative phosphorylation, amino acid metabolism, and mTOR pathways, indicating programming for the high energy utilization and anabolism necessary for rapid expansion of the population. There has been much focus on cancer cell–intrinsic genetic and epigenetic programs that facilitate the adaptation of their metabolisms to allow proliferation in nutrient-scarce microenvironments and to out-compete the nontransformed cells in the TME. Under conditions of limited glucose, cells can reprogram their metabolism toward other pathways, including oxidative phosphorylation and mTORC1 signaling, to drive energy generation (44, 45). Under lower oxygenation, tumor cells activate cellular stress responses to mitigate the negative effect of oxygen deprivation and preserve proliferative capacity. Notably, these same pathways are also among the highly induced pathways in the MDSC population (in particular E-MDSC) from our GSEA analysis. There is also emerging evidence in other cancers that metabolic reprogramming of MDSCs can play an important role in their immunosuppressive and protumoral activities. The metabolic pathways identified from our results provide insights into additional mechanisms by which MDSCs can promote T cell suppression and tumor growth in glioblastoma and can lay the groundwork to harness myeloid cell–directed immunometabolic therapies.

In addition to up-regulation of additional metabolic pathways, to maintain their proliferative expansion and evade apoptosis to thrive in the harsh hypoxic environment of the pseudopalisading region, E-MDSCs also reprogram their transcriptomes to handle hypoxia and oxidative stress, highly expressing metallothionein genes (*MT2A* and *MT1G*) and genes involved in heme degradation (*HMOX1*, *FTL*, and *FTH1*). All of these programs are hallmarks of tumor-intrinsic biology but have not previously been appreciated to be highly expressed in and functionally relevant for geographically localized MDSC populations. Metallothioneins function as potent antioxidants through scavenging of free radicals and have been associated with increasing glioma grade and worse survival (46). *HMOX1* is the rate-limiting enzyme in the catabolism of free heme and plays a key role in regulating anti-inflammatory and antioxidant pathways. Our results indicate that MDSCs have similar compensatory mechanisms to actively reprogram their metabolism to ensure survival in the hypoxic TME, and these pathways represent additional weaknesses in MDSCs that can be exploited in the generation of myeloid-targeted therapies.

The cellular components of the IDH-WT glioblastoma immune microenvironment have often been viewed in a static state of terminal differentiation. Using computational lineage tracing, we discovered that the myeloid cells in the TME represent a dynamic continuum of cellular states. Furthermore, cells in immature states, such as E-MDSCs, have the potential to transition into M-MDSCs. This finding indicates that not only do E-MDSCs have the capacity to promote tumor progression and immune suppression, but they also have the capability to transition into other cellular states that also have the ability to promote tumor growth and T cell suppression through distinct but complementary mechanisms. This suggests that targeting one population of MDSCs might be suboptimal in counteracting their effect on glioblastoma progression, whereas targeting multiple populations is required to effectively address the immunosuppressive IDH-WT glioblastoma TME.

Although IDH-WT glioblastoma rarely metastasizes systemically, it remains one of the most recalcitrant tumors owing to its aggressive invasion and infiltration into surrounding normal brain tissue—which makes surgical cure impossible—with recurrence inevitable despite treatment. Prior studies have suggested that a hypercellular zone of cells migrating away from a necrotic focus, defined pathologically by the pseudopalisading region of IDH-WT glioblastoma, may be responsible for the highly invasive nature of this malignancy. Although prior studies have shown that the cells in this zone secrete high levels of proangiogenic factors that promote tumor growth (20), the origins of these factors have not been defined. Previous studies have assumed that the pseudopalisades are mostly made up of migrating tumor cells with very limited evaluation of the presence of any immune cell components in this region (24). It was not until more recently that the potential roles of immune cells in the pseudopalisading region was described. Saavedra-López *et al*. demonstrated the presence of glioma-associated microglia and macrophages that migrate toward the necrotic core in the setting of hypoxia and are involved in debris clearance (47). However, limited cellular markers were used to fully characterize the phenotypes of these GAMs. In glioma mouse models, hypoxia niches have been demonstrated as a potential driver of GAM immunosuppression (48). Our data represent the first large-scale single-cell characterization of the pseudopalisading region of IDH-WT glioblastoma. Our scRNA-seq and spatial transcriptomic results revealed that the pseudopalisading region is mainly composed of MDSCs and a specific tumor cell subset with gene expression previously demonstrated in cancer cells with stem-like and mesenchymal programs. We hypothesize that this hypoxic core leads to tumor cell migration and microvascular proliferation at its periphery, resulting in the recruitment of MDSCs from the peripheral blood to the TME. Our data demonstrate that the tumor cell population with mesenchymal and stem-like programs likely supports the development and trafficking of the MDSCs through chemokines signaling, such as *CSF1-CSF1R*, *CCL5-CCR1*, and *CCL8-CCR1*. And, reciprocally, the MDSCs potentially support the growth and invasion of the glioma cells with stem-like features in the hypoxic environment through engagement of growth factor (*FGF11-FGFR1* and *VCAN-EGFR*) and cellular migration (*FN1-CD44*) pathways. We describe an interaction between FGF11 and FGFR1 previously not elucidated in IDH-WT glioblastomas that potentially drives tumor growth and invasion. FGFR1 has been demonstrated to play critical roles in tumor growth and stemness in other cancers (49). In glioblastoma, it has been shown to be associated with invasive tumor cells and radioresistance (50, 51). There are 18 ligands that engage with the FGFR family, with the role of FGF11 being reported in only a limited series of studies. Overexpression of FGF11 has been demonstrated to be associated with worse prognosis and immunosuppression in lung adenocarcinoma (52, 53). It has also been found to promote tumor aggression in breast cancer, ovarian cancer, and oropharyngeal carcinoma (54–56). Our study demonstrates its role in glioblastoma, in potentially promoting tumor invasion and tumor stemness, which can represent a potential therapeutic target for these patients.

Recently, Venkataramani *et al*. (57) have proposed that the oligodendrocyte progenitor–like (OPC) and neural progenitor–like (NPC) tumor cells, as defined by Neftel *et al*. (12), are the critical cell types that drive tumor invasion by hijacking neuronal mechanism. Although we did identify tumor cells with OPC and NPC meta-modules within our tumor clusters, they are distinct from the T4 cluster and localize to separate geographical areas from the pseudopalisading region, where the E-MDSC and T4 reside. We propose that the T4 population at the pseudopalisading perinecrotic border with a stemness-associated genetic program is a driver of growth and aggressiveness of the tumor and that this is a separate process from the neural invasion process at the invasive edge as described by Venkataramani *et al*. (57). The potential relationship between the T4 population and the OPC and NPC populations, including the temporal kinetics in the generation of these functionally distinct tumor populations, is worth additional investigation in subsequent studies.

Our spatial transcriptomic data further corroborate the colocalization of E-MDSC and tumor cell populations with mesenchymal and stem-like programs and enriched expression of the canonical ligand-receptor pairs to the pseudopalisading region. This phenomenon is exclusively observed in IDH-WT glioblastomas compared with their counterpart IDH-mutant grade 4 astrocytomas. Our results further suggest that this observed difference is potentially associated with epigenetic regulation of the expression of key chemokines responsible for MDSC recruitment in IDH-WT tumors.

Our study provides valuable insights into the role of glioblastoma-specific MDSCs in human IDH-WT glioblastoma; however, its scope for functional exploration is currently limited by the absence of models that faithfully recapitulate the intricate myeloid-tumor cell interactions and landscape observed in human tumors. Commonly used murine models, though informative, notably lack the presence and function of E-MDSCs—the critical subset of myeloid cells highlighted in our study. Furthermore, human glioblastoma organoid systems fall short in this regard because they exhibit insufficient immune cell infiltration. Recent research underscores their inability to model hypoxia-induced tumor phenotypes, a crucial aspect of glioblastoma TME, particularly in the pseudopalisading regions central to our investigation. Our spatial transcriptomic analysis partially mitigates these limitations by revealing a clear colocalization of E-MDSCs and T4 cells to the pseudopalisading region. Moreover, it demonstrates proximity of cognate chemokines, growth factors, and receptors associated with these cells in the same area. Complementing this, our profiling of hundreds of glioma samples from TCGA confirmed the exclusive presence of these cells in the most aggressive IDH-WT glioblastomas. We also found that the presence of E-MDSCs and T4 cells serves as an independent prognosticator of overall survival, even within the IDH-WT glioblastoma subgroup. These findings suggest a potential role for E-MDSCs in promoting glioblastoma aggressiveness, likely through their interactions with tumor cells exhibiting stem-like programs. In the future, it will be critical to develop experimental models that recapitulate this critical tumor-immune interaction to mechanistically dissect it and also to test new therapeutic approaches that disrupt it.

Our large-scale single-cell analysis elucidated distinctive MDSC populations as potential facilitators of IDH-WT glioblastoma progression and mediators of tumor immunosuppression. Our results elucidate the phenotypic and functional heterogeneity of MDSC populations in glioblastoma with each population exhibiting specific gene signatures and phenotypic markers that differentiate them from other BMDMs. We further illuminated the possible molecular mechanisms that govern the specific recruitment and metabolic programs that allow the accumulation of MDSCs in the IDH-WT glioblastoma TME along with specific ligand-receptor interactions between MDSCs and glioma cells that likely contribute to their potential role in promoting tumor invasion. Finally, we introduce insights into the aggressive and invasive pseudopalisading region that is a hallmark of glioblastoma, with evidence that MDSCs, alongside a population of glioma cells with stem-like and mesenchymal features, contribute significantly to the composition of the pseudopalisades. Notably, these MDSC populations were not seen in IDH-mutant gliomas, likely associated with DNA hypermethylation and silencing of the key chemokines responsible for their recruitment. Our comprehensive analysis lays the foundation for the development of new strategies to target the recruitment of these specific MDSCs, their metabolism, and tumor-promoting mechanisms as promising therapeutic intervention in this deadly cancer.

## Materials and methods

### Human subjects

Patients were prospectively enrolled into this study at Johns Hopkins Hospital. Written informed consent was provided by all participants according to approved Institutional Review Board Protocol. Fresh tumor tissue and blood were collected at the time of surgery after confirmation of primary glial neoplasm on frozen section. None of the patients was treated with chemotherapy or radiation before tumor resection. Tumor samples span all glioma grades and subtypes. Tumors were categorized according to the 2021 WHO Classification of Central Nervous System Tumors based on the combination of relevant histopathologic and molecular features. Patients undergoing anterior temporal lobectomy for seizure control were consented and nonneoplastic tissue from anterior temporal lobectomy resection was collected as control.

### Tumor dissociation

Patient tumors or nonmalignant brain tissue were mechanically cut into about 1-mm^3^ tissue in Dulbecco’s modified eagle medium (DMEM) (Gibco). They were then enzymatically digested with the human tumor dissociation kit (Miltenyi Biotec) on the gentleMACS Dissociator (Miltenyi Biotec) per manufacturer instructions. Dissociated cells were filtered by a 70-mm strainer and centrifuged at 300*g* for7 min. After removing the supernatant, the pelleted cells were suspended in red blood cell lysis buffer (Miltenyi Biotec) for 2 min to remove red blood cells. Tumor samples with significant extent of myelin underwent myelin removal process using magnetic Myelin Removal Beads (Miltenyi Biotec) on the autoMACS (Miltenyi Biotec). The cells were then washed with sorting buffer [phosphate-buffered saline (PBS) supplemented with 2% fetal bovine serum (FBS)] and accessed for viability using trypan blue exclusion.

### PBMC collection

PBMCs were isolated after Ficoll-Paque centrifugation. Briefly, 80 ml of fresh peripheral blood was collected at the time of surgery in EDTA anticoagulant tubes and layered on top of Ficoll-Paque after twofold dilution with PBS. After centrifugation (2200 rpm, 20 min, no brake), PBMCs are collecting from the mononuclear cell bandlayer. PBMCs are viability frozen in 10 million per milliliter until future use.

### FACS

Single-cell suspensions of the tumor and non-neoplastic brain tissue were stained with antibodies for 30 min at 4°C against CD45+ (PE, BD Bioscience), CD3+ (APC, BD Bioscience) and nuclear stain DyeCycle Violet (Invitrogen) for FACS, performed on Beckman Coulter MoFlo XDP. Cells were sorted into three live populations, T cells (CD45+CD3+), non-T immune cells (CD45+CD3−), and nonimmune cells (CD45−, DyeCycle Violet+) directly into cold PBS + 1% bovine serum albumin (BSA) (Sigma-Aldrich) with a post-sort purity of >98%. Sorted cells were then counted and assessed for viability using trypan blue and resuspended in 700 cells/μl in PBS + 0.04% BSA.

### scRNA-seq and processing

scRNA-seq of myeloid and tumor cells was performed using the 10X Single Cell 3′ Gene Expression Kit v3, and scRNA-seq and single-cell T cell receptor sequencing (scTCR-seq) of T cells were performed using the 10X Single Cell 5′ Immune Profiling Kit v1.1 (10X Genomics, Pleasanton, CA, USA). Cells were captured in droplets at a targeted cell recovery of 5000 to 10,000 cells per lane. scRNA library generation was performed per protocol. After cell barcoding in droplets and reverse transcription, emulsions were broken and cDNA purified using Dynabeads. cDNA was amplified by 11 polymerase chain reaction (PCR) cycles for 3′ gene expression and 13 PCR cycles for 5′ immune profiling. Amplified cDNA was then used for 3′ gene expression library construction or 5′ gene expression library construction and TCR enrichment. Sequencing was performed using an Illumina NovaSeq 6000 with 310 million reads per sample and a sequencing configuration of 26 × 8× 98 (UMI × Index × Transcript read). The Cell Ranger 3.0.2. pipeline software (10X Genomics, California) was used to align reads and generate expression matrices for downstream analysis.

### scRNA-seq filtering and normalization

Lymphoid, myeloid, and tumor datasets were processed separately. After combining raw counts from the filtered output of CellRanger, we further filtered cells based on both minimum number of genes and counts. Selection of each was performed using histograms of each parameter by cell (fig. S1A). In each case, a minimum threshold of 500 genes and 750 unique molecular identifier (UMI) counts was sufficient to exclude populations of low-quality cells visible as the lower portion of the bimodal/multimodal distributions visualized in the histogram. Genes expressed in fewer than 0.1% of the cells were also removed. Finally, counts were normalized to the total UMI count by cell and log-scaled using Seurat.

### Data integration

Data integration was performed using Seurat v3 using reciprocal principal components analysis (PCA) to project cells into a shared space followed by identification of anchors for mutual nearest neighbor integration. One female and one male patient were selected as references for integration.

### Scaling, PCA, clustering, and dimensional reduction

Scaling of both corrected and uncorrected normalized gene expression values was calculated, although the uncorrected values were used only for visualization of differential gene expression with heatmaps. In both cases, values were scaled to a mean of zero and a standard deviation of one by gene. The scaled, corrected values were used for PCA, after which 60 principal components were selected based on elbow plot for all datasets. The 60 principal components were used for dimensional reduction by UMAP as well as generation of a shared nearest neighbor network followed by Louvain clustering. Clusters with very similar gene expression profiles were combined and in some cases, clusters were isolated for further clustering.

### Differential gene expression

Library size normalized gene expression matrix was imputed using SAVER (58) to address potential dropouts, and the imputed values are log_2_-transformed after adding pseudocount of 1. A linear mixed-effect model is used to identify genes that are significantly differential between cell clusters or between two groups of samples within each cell cluster. The details of the mixed-effect model can be viewed at the following GitHub page: https://github.com/zji90/Raisin/. The *P* values are adjusted for multiple testing using the Benjamini-Hochberg procedure. Genes with false discovery rate (FDR) < 0.05 are considered to be significant differential genes.

### Cell cycle scoring

Seurat’s CellCycleScoring was used to identify cells with high G2M or S phase contributions. Although this was not used for regression of gene expression values during scaling of all cells, it was used to identify clusters of cells undergoing mitosis. These clusters were isolated and then scaling was performed on these cells alone with regression of cell cycle scores. Finally, PCA and clustering were performed on the regressed, scaled values to attempt to identify types of cells present in the cycling clusters.

### MDSC suppression assay

MDSCs were isolated from glioma tumors using FACS. Tumor-associated myeloid cells were harvested through tumor dissociation. Surface markers were directly stained with the following fluorochrome-conjugated antibodies (table S3). Cell viability was assessed using propidium iodide (Stem Cell Technologies). Cells were sorted using MoFlow XDP directly into cold RPMI supplemented with 10% heat-inactivated FBS (ThermoFisher) and 10 mg/ml gentamicin (ThermoFisher). Cells were then washed, counted, and resuspended at concentration for suppression assay.

CTV-labeled responder PBMCs from a healthy donor were cocultured with different effector-to-target ratios with unlabeled tumor MDSCs sorted from patients with glioma. T cell proliferation was induced by addition of anti-CD3/anti-CD28 coated microbeads (Dynabeads Human T Activator, ThermoFisher) at a bead-to-cell ratio of 1:4. Cells were cultured for 96 hours in 96-Ubottom plate in complete RPMI medium (Gibco). Proliferation was assessed using CTV dilution detected by a Celesta flow cytometer. Relative proliferation was calculated against stimulated responder PBMC that were cultured without MDSC. Data are representative of this assay done multiple times with different patients all displaying similar results.

### Calculation of transcription factor signaling networks

The SCENIC workflow was first used to identify transcription factor activities by cell. All datasets were pooled and then 50,000 cells sampled from the target metadata due to memory limits. Transcription factor scores were then correlated with receptor expression after exclusion of receptors which were predicted targets of specific transcription factors. These correlations were used to build a network between transcription factor and receptor. Finally, ligand pairs for predicted receptors were identified using CellphoneDB2. After generation of networks for high- and low-grade tumors, the two networks were compared to identify transcription factors and receptors predicted to be activated in only high- or low-grade tumors.

### RNA velocity analysis

RNA velocity analysis was performed as previously described using the velocyto.py.python package for annotating transcripts as spliced or unspliced, followed by the velocyto.R R package to perform velocity estimation. Briefly, transcripts are marked as either spliced or unspliced based on the presence or absence of intronic regions in the transcript. For each gene, a simple model of RNA dynamics is then fit to the data. Lastly, the RNA velocity is estimated for each cell by looking for over- or underrepresentation of spliced to unspliced ratios. RNA velocity is visualized on a diffusion plot, with vector fields representing the averaged velocity of nearby cells (59).

### Pseudotime analysis

We apply TSCAN (v.1.7.0) to reconstruct the pseudotime on diffusion map space for the cells of six cell types (E-MDSC, M-MDSC, MAC1, MAC2, PMN-MDSC, and NEUT) from IDH-WT glioblastoma patients. Our method identifies genes or transcripts that vary along a pseudotime trajectory with statistical significance.

We consider the gene expression pattern along a pseudotime trajectory as functional data and represent the data using B-spline basis. We describe the expression of gene *g* along pseudotime t=1,…,T in a hierarchical model. The first hierarchy is to describe the population-level pattern as $x_{s}^{T}{\text{β}}_{ig}$, where $x_{s}$ is the design matrix for sample s, and ${\text{β}}_{ig}$ is the coefficient with reference to the i-th B-spline basis and gene g. To test whether a gene variates along a pseudotime trajectory, we let $x_{s}=I_{1}$ and ${\text{β}}_{ig}\in R$. It is straightforward to see that the population pattern on all basis is $X_{s}^{T}{\text{β}}_{g}$, where $X_{s}=I_{N}⊗x_{s},{\text{β}}_{g}={\left[{\text{β}}_{1g},{\text{β}}_{2g},\ldots ,{\text{β}}_{Ng}\right]}^{T}\in R^{N}$, and N is the number of B-spline basis. The second hierarchy is to describe the sample-level pattern as $x_{s}^{T}{\text{β}}_{ig}^{s}+u_{igs}:=a_{igs}$, where $u_{gs}\sim N\left(0,\tau_{g}\right),\forall i,a_{igs}\sim N\left(x_{s}^{T}{\text{β}}_{ig},\tau_{g}\right)$. The third hierarchy is to describe the observed expression of gene g in cell c of sample s as $e_{gcs}=\sum_{i=1}^{N}\varphi_{i}\left(t_{gcs}\right)a_{gs}+\epsilon_{gcs}$, where $\epsilon_{gcs}\sim N\left(o,\sigma_{gs}^{2}\right),\forall c$.

The parameters are $\theta =\left(\tau_{g},\sigma_{gs}^{2},{\text{β}}_{g}\right)$, where $\tau_{g}\in R^{S\times S},\sigma_{gs}\in R,{\text{β}}_{g}\in R^{N},S$ is the number of samples (patients), the latent variable is $u_{gs}\in R^{N}$, and $\phi_{cs}\in R^{N\times 1}$ is the B-splines basis function of N basis.

We apply expectation-maximization (EM) algorithm to solve this problem and obtain the estimated ${\text{β}}_{g}$ for further statistical testing. We perform permutation test where we permute the pseudotime order of the cells and bootstrap the cells within each sample 100 times. In each permutation, we rerun the above model and redo EM. The number of knots (range from 0 to 30) are automatically selected using BIC. Maximal EM iteration is 100. Convergence cutoff is 1. The null is that all elements in ${\text{β}}_{g}={\left[{\text{β}}_{1g},{\text{β}}_{2g},\ldots ,{\text{β}}_{Ng}\right]}^{T}\in R^{N}$ are 0. We consider the P value as the percentage of permutation (out of 1000 times) that have log-likelihood greater than the original (pseudotime is not-permuted) log-likelihood. We apply the Benjamini-Hochberg (BH or its alias FDR) method to adjust multiple testing. We consider genes with adjusted P<0.05 as trajectory differential with statistical significance. We apply k-means clustering to group trajectory differential genes with similar pseudotime pattern using standardized gene expression levels. The averaged model-fitted values (fitted resolution = 1000) on standardized scale of the genes within each cluster are used to represent the cluster pattern (60).

### Immune-metabolic ex vivo flow cytometry staining

All flow cytometry antibodies used for phenotypic and metabolic analysis can be found in table S4. Cryopreserved PBMCs or tumor single-cell suspensions were thawed in RPMI (Gibco) + 20% FBS (Atlanta Biologicals). Cells were washed once in PBS and immediately stained for viability with Biolegend Live/Dead Zombie NIR fixable Viability Dye and BD Fc Block for 10 min at room temperature. Cell surface staining was performed in 100 μl of 20% BD Horizon Brilliant Stain Buffer + PBS with surface stain antibody cocktail for 20 min at room temperature. Cells were washed with 1X permeabilization/wash buffer. Intracellular staining (ICS) was performed in 100 μl of 1X permeabilization/wash buffer with ICS antibody cocktail for 45 min at room temperature. Cells were washed once with permeabilization/wash buffer, then resuspended in 1% paraformaldehyde for acquisition by flow. Samples were run on a 3 laser Cytek Aurora spectral flow cytometer. FCS files were analyzed using Flowjo v10 (10.6.2.) software. High-dimensional unbiased analysis of cell phenotypes was performed using FlowJoDownsample v3 and UMAP.

### Cell-cell interaction analysis and ligand-receptor interaction analysis

#### Pseudobulk gene expression data

We first obtained the pseudobulk gene expression matrix for each patient as follows: aggregate the raw read count within each cell cluster, normalized the data by count per million (CPM), then log_2_-transformed the data after adding pseudocount 1. Pseudobulk gene expression matrices are generated with each matrix corresponding to a patient sample. Within each pseudobulk matrix, each row represents a gene and each column represents a cell type.

#### Normalized interaction score

We used a curated list of immune-related and non–immune-related ligand-receptor pairs from previously published literature (table S5). The normalized interaction score for a specific ligand-receptor pair between cell type A and cell type B is defined as the product of min-max normalized pseudobulk expression levels of ligand in cell type A and that of receptor in cell type B.


$$NIS\;\left(\begin{array}{c} \;ligand\text{,}\;cell\;type\;A,\\ \;receptor\text{,}\;cell\;type\;B \end{array}\right)=\;\;norm\_pb\_exp\text{r}\;(ligand\text{,}\;cell\;type\;A)\;\;\times \;norm\_pb\_expr\;(receptor\text{,}\;cell\;type\;B)$$

where NIS is the normalized interaction scores; *norm_pb_expr*(*ligand*, *cell type A*) is the min-max normalized pseudobulk expression levels of ligand in cell type A; and *norm_pb_expr* (*receptor*, *cell type B*) is the min-max normalized pseudobulk expression levels of receptor in cell type B.

Specifically, for each patient sample, we first divided the pseudobulk gene expression matrix into three subsets based on the cell population—i.e., myeloid, tumor, and T cells. We then performed the min-max normalization across each gene within each cell population. Through this normalization, for each gene, we assigned a value of 1 to the cell type with the highest expression level and a value of 0 to the cell type with the lowest expression level. This normalization method allowed us to identify ligand and receptor pairs that were highly expressed in specific cell types within the larger cell population, rather than those genes that were universally highly express across all cell types.


$$f\left(x_{gijk}\right)=\frac{x_{gijk}-\underset{1\leq l\leq K_{ij}}{\min}\left(x_{gijl}\right)}{\underset{1\leq l\leq K_{ij}}{\max}\left(x_{gijl}\right)-\underset{1\leq l\leq K_{ij}}{\min}\left(x_{gijl}\right)}$$

Here, f is the min-max normalization function and $x_{gijk}$ is the pseudobulk expression levels of gene g, patient sample i, cell population j, and cell type k. $K_{ij}$ is the number of cell types within patient sample i and cell population j, and min and max operations are iterated over all possible cell types $1\leq l\leq K_{ij}$.

#### Identifying significant cell clusters and ligand-receptors

We searched through all possible cell cluster pairs—i.e., pairwise combinations between 16 T cell clusters, 10 tumor cell clusters, and 18 myeloid cell clusters. For each cluster pair, we summarized the number of significant ligand-receptor pairs whose interaction score could differentiate IDH-WT glioblastoma patients from IDH-mutant tumors. For each ligand-receptor pair, Wilcoxon rank-sum test was performed to compare the normalized interaction scores between IDH-WT glioblastoma patients and IDH-mutant glioma patients. We also defined the enrichment score as the difference between average normalized interaction scores in IDH-WT glioblastoma patients and IDH-mutant glioma patients. Benjamini-Hochberg method was applied to adjust for multiple testing, and ligand-receptor pairs with FDR < 0.05 and enrichment score > 0.3 were reported as significant.

#### Ligand-receptor cross-sample correlation analysis

For each cell cluster pairs of interest, we also detected ligand-receptor pairs whose expressions were highly correlated across IDH-WT glioblastoma patients. Spearman correlation was calculated and circle plots were made using the *circlize* package (61).

#### TCGA correlation analysis

The TCGA glioblastoma gene expression RNA-seq count data were downloaded from UCSC Xena, which included 173 samples. For each sample, we defined the gene signature scores for a cell cluster by the average expression values of top differentially expressed genes (FDR< 0.05 and log_2_ fold change > 0.5). We then calculated the Pearson correlation between signature scores of myeloid cell clusters and T cell clusters, as well as myeloid cell clusters and tumor cell clusters. To account for the potential effect of CD45+ cell levels, we also calculated the adjusted gene signature scores—i.e., dividing the original signature scores by the expression levels of *PTPRC* (CD45).

#### Correlation of receptor level with induced gene set programs

Genes associated with signaling of cognate receptors identified from ligand-receptor interaction analysis were identified using established gene sets from Molecular Signatures Database. These were used to generate cell level scores with UCell (62). The resulting scores were linearly correlated (Pearson correlation) with normalized RNA expression for the paired receptor and visualized with a pseudocolor plot.

### Visium spatial transcriptomics experiment

Formalin-fixed paraffin-embedded (FFPE) blocks of surgically resected glioblastoma tissue from two subjects were cut and sectioned into both hematoxylin and eosin (H&E)–stained slides and unstained slides. H&E slides were reviewed by one of the authors (C.-H.L.) to confirm the diagnosis and annotate regions of interest within a 6.5-mm–by–6.5-mm area. One unstained section from each tissue sample underwent deparaffinization and de–cross-linking. Stained slides were scanned using the VENTANA DP 200 platform (Roche) to preserve high-quality tissue images. Probe hybridization and subsequent steps including analyte transfer to Visium slides were conducted using Visium CytAssist Spatial Gene Expression (CG000495, 10X Genomics) following the manufacturer’s instructions. In brief, probe hybridization was performed overnight, followed by probe ligation and extension, with involvement of CytAssist instrument to release and transfer the probe from tissue slides to Visium slides. After cDNA amplification, spatially barcoded libraries were constructed and sequenced on the Illumina NovaSeq 6000 platform to achieve a depth of at least 25,000 mean read pairs per spot.

#### Visium spatial transcriptomics data processing

The Space Ranger pipeline was used to process the Visium spatial gene expression data. This pipeline enabled probe alignment to 10X Visium Human Transcriptome Probe Set v2.0, and generated feature barcode matrix and spatial location data for downstream analysis. The resulting count matrix and associated H&E images were then used by the R package Seurat (v5.0) for spatial transcriptomic analysis (63). SCTransform was performed for sample normalization, followed by PCA, dimensionality reduction and clustering. Differential expression analysis of each cluster was conducted by the “FindAllMarkers” function in Seurat.

#### Cell type deconvolution by RCTD

Our scRNA-seq data were used as reference to determine the cell type composition of each Visium spot by RCTD (26). RCTD objects were created for each sample, with “max_cores = 8.” The RCTD pipeline was run with parameter “doublet mode = full” to enable estimating for more than two cell types. The results were normalized to cell type weights for each spot.

#### Cell proportion representation as a function of distance from necrosis

Nonnecrotic spots in the Visium data were stratified into 10 regions based on Euclidean distance from necrotic spots. The proportion of spots with high E-MDSC, T3, and T4 cell infiltration (greater than median deconvolution for each cell type) were plotted as a function of the first five regions closest to necrotic spots.

#### Spatially informed ligand-receptor cross-sample correlation analysis

Given the inherent smoothness of spatial transcriptome, we apply a smoothing step to the spot-level gene expression by averaging its adjacent neighbors to improve the signal-to-noise ratio of data. We assume that, for a ligand-receptor pair, the spot-level receptor gene’s expression could be affected by the ligand gene’s expression in the spot’s niche—i.e., its microenvironment. We define the niche-ligand expression by the average expression of the ligand within spots from 20 nearest neighbors. In this way, a one-to-one match is established between the spot-receptor expression and niche-ligand expression, where the adjusted $R^{2}$ (coefficient of determination) statistic is used to measure their correlation. The spatial information is implicitly incorporated as an add-on to the pseudobulk correlation analysis.

#### Define and compare perinecrotic versus distant region ligand and receptor correlation

The necrotic, pseudopalisading (perinecrotic), and distant tumor regions were annotated by a board-certified neuropathologist. Given the differences in the architecture of the two patient samples, for the purpose of the ligand and receptor correlation analysis, we further defined the perinecrotic regions as the union of top 50% closest spots to the necrotic regions (excluding the necrotic regions themselves). The rest of the sample, other than necrotic and perinecrotic regions, is defined as distant regions. We calculate the adjusted $R^{2}$ statistics for ligand-receptor pairs for perinecrotic and distant regions separately. The presence of significant differences between the $R^{2}$ in these two regions suggests region-specific gene versus gene interaction and the potential underlying mechanism.

## Funding:

This research was supported by National Institutes of Health (NIH) grant F32NS108580 (C.J.), the Neurosurgery Research Education Foundation (C.J.), funding through the Bloomberg~Kimmel Institute for Cancer Immunotherapy (D.P. and J.E.), NIH grant R01HG010889 (H.J.), NIH grant R01HG009518 (H.J.), Burroughs Wellcome Career Award for Medical Scientists (C.B.), NIH grant RA37CA230400 (C.B.), NIH grant U07CA230691 (C.B.), the Commonwealth Foundation (S.Y.), the Maryland Cigarette Restitution Fund (S.Y.), and the NIH Pioneer Award (J.E.).

## Supplementary Material

Supplementary Materials

S2,S5,S6

the MDAR Reproducibility Checklist

## Data Availability

Raw and processed scRNA-seq and spatial transcriptomics data are available at the NCBI’s Gene Expression Omnibus (GEO) (accession nos. GSE278456 and GSE276841). All other data are available in the main text or the supplementary materials. Custom computational methods beyond routine algorithms and packages are publicly accessible through Zenodo (64).
