# Supplementary material for: Distinct myeloid-derived suppressor cell populations in human glioblastoma
Source: Science. Author manuscript; Available in PMC 2026 Jan 27. (PMC12836367; doi:10.1126/science.abm5214)
Supplement: Supplementary Materials [file NIHMS2115262-supplement-Supplementary_Materials.pdf]

Science
AAAS

Supplementary Materials for

Distinct myeloid-derived suppressor cell populations in human glioblastoma

Christina Jackson *et al*.

Corresponding authors: Christina Jackson, christina.jackson2@pennmedicine.upenn.edu; Srinivasan Yegnasubramanian, syegnasu@jhmi.edu; Drew Pardoll, dpardol1@jhmi.edu

Science **387**, eabm5214 (2025)
DOI: 10.1126/science.abm5214

The PDF file includes:

Figs. S1 to S10
Tables S1, S3, and S4

Other Supplementary Material for this manuscript includes the following:

Tables S2, S5, and S6
MDAR Reproducibility Checklist

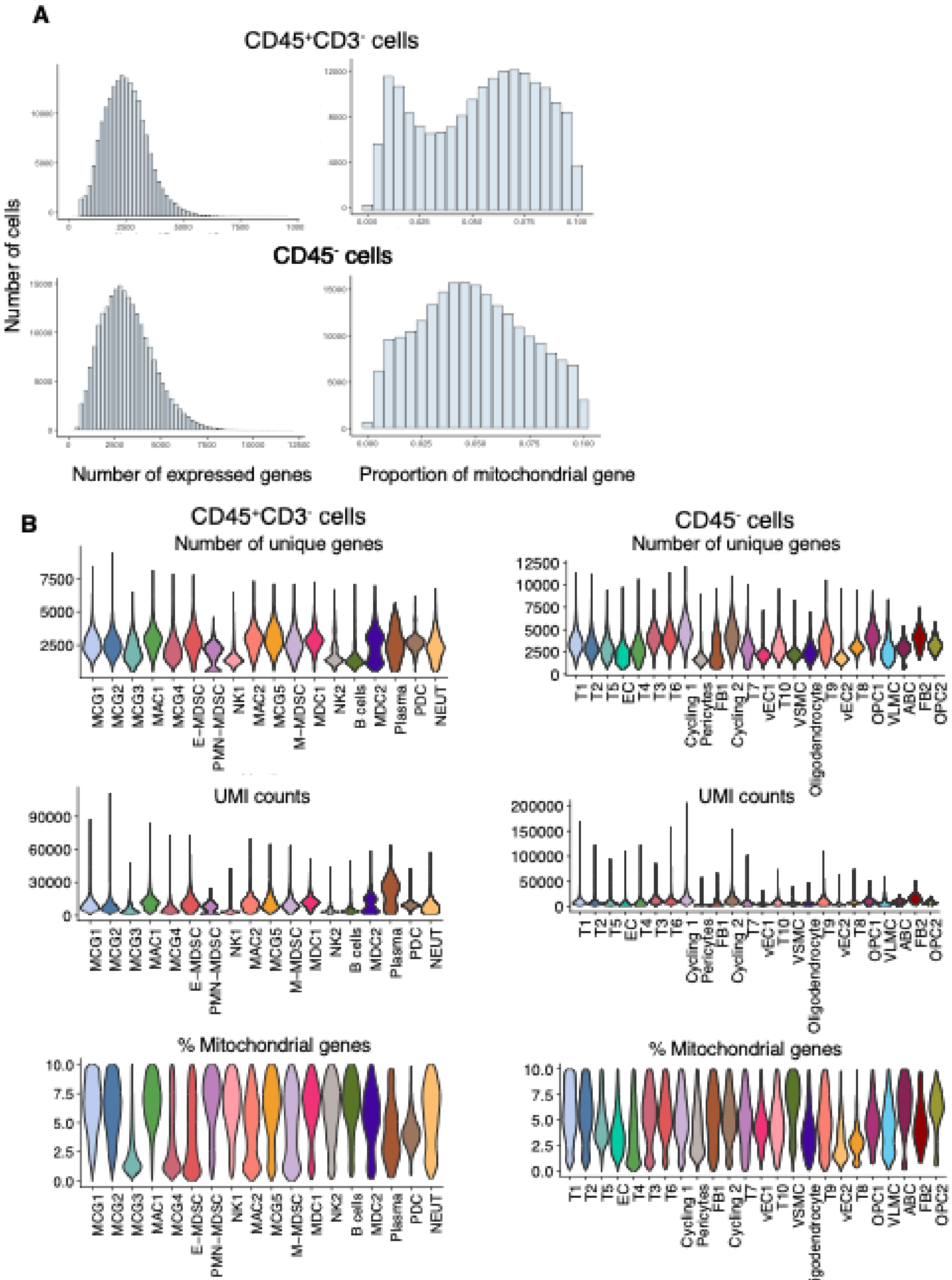
A
CD45+CD3- cells
CD45- cells
Number of cells
Number of expressed genes
Proportion of mitochondrial gene
B
CD45+CD3- cells
Number of unique genes
UMI counts
% Mitochondrial genes
MCG1
MCG2
MCG3
MAC1
MCG4
E-MDSC
PMN-MDSC
NK1
MAC2
MCG5
M-MDSC
MDC1
NK2
B cells
MDC2
Plasma
PDC
NEUT
CD45- cells
T1
T2
T5
EC
T4
T3
T6
Cycling 1
Pericytes
FB1
Cycling 2
T7
vEC1
T10
VSMC
Oligodendrocyte
T9
vEC2
T8
OPC1
VLMC
ABC
FB2
OPC2

fig. S1. Quality control of tumor and myeloid cells

(A) Histograms demonstrating the distribution of genes captured and proportion of mitochondrial related genes across $CD45^{+}CD3^{-}$ (top) and $CD45^{-}$ (bottom) cells. (B) Violin plots demonstrating the distribution of number of unique genes (top), total UMI counts (middle), and percentage of reads that map to the mitochondrial genome (bottom).

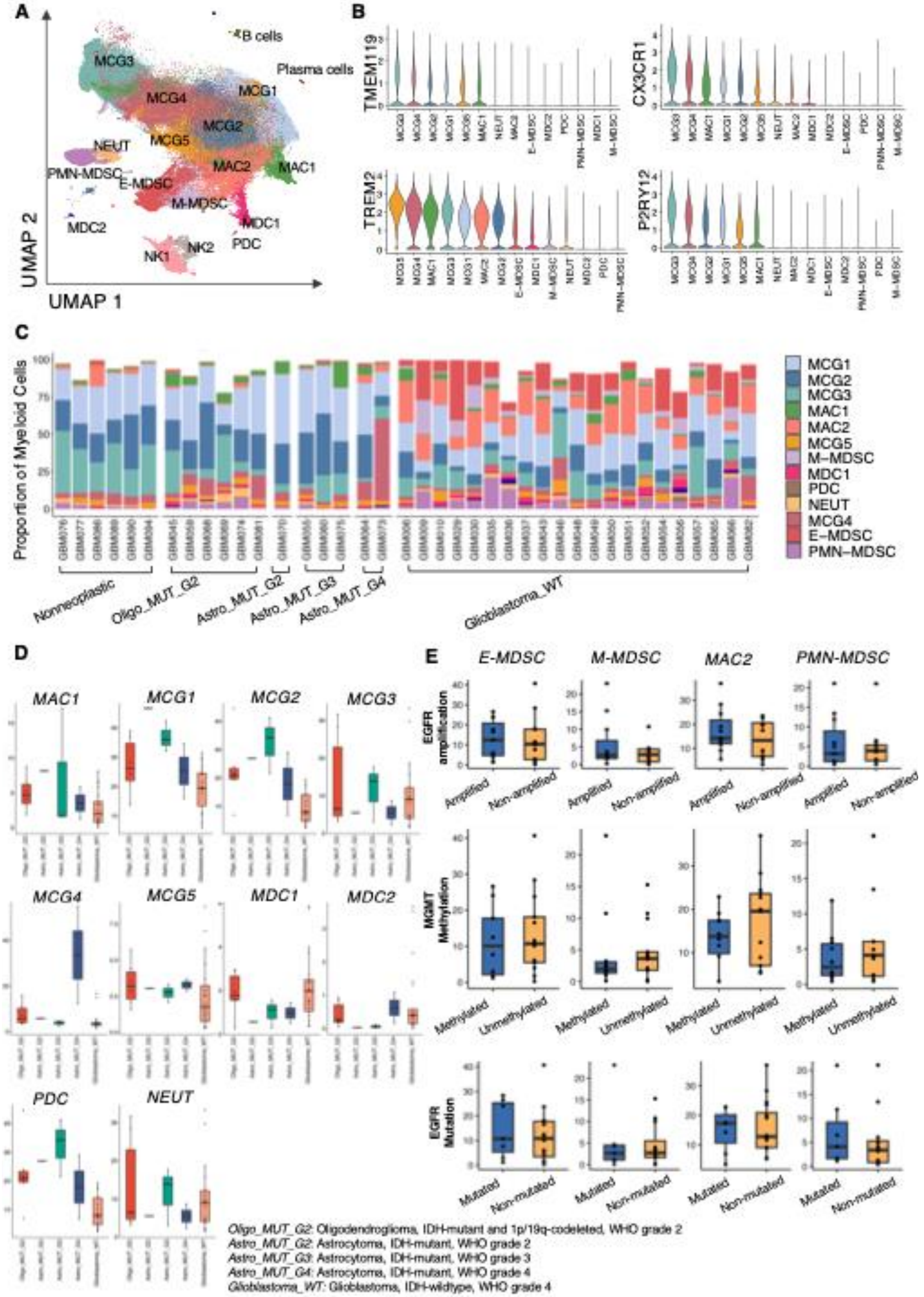
A
B cells
Plasma cells
MCG3
MCG4
MCG1
MCG2
MCG5
NEUT
MAC2
MAC1
PMN-MDSC
E-MDSC
M-MDSC
MDC1
MDC2
PDC
NK1
NK2
UMAP 2
UMAP 1
B
TMEM119
CX3CR1
TREM2
P2RY12
C
Proportion of Myeloid Cells
MCG1
MCG2
MCG3
MAC1
MAC2
MCG5
M−MDSC
MDC1
PDC
NEUT
MCG4
E−MDSC
PMN−MDSC
Nonneoplastic
Oligo_MUT_G2
Astro_MUT_G2
Astro_MUT_G3
Astro_MUT_G4
Glioblastoma_WT
D
MAC1
MCG1
MCG2
MCG3
MCG4
MCG5
MDC1
MDC2
PDC
NEUT
E
E-MDSC
M-MDSC
MAC2
PMN-MDSC
EGFR amplification
Amplified
Non-amplified
MGMT Methylation
Methylated
Unmethylated
EGFR Mutation
Mutated
Non-mutated
Oligo_MUT_G2: Oligodendroglioma, IDH-mutant and 1p/19q-codeleted, WHO grade 2
Astro_MUT_G2: Astrocytoma, IDH-mutant, WHO grade 2
Astro_MUT_G3: Astrocytoma, IDH-mutant, WHO grade 3
Astro_MUT_G4: Astrocytoma, IDH-mutant, WHO grade 4
Glioblastoma_WT: Glioblastoma, IDH-wildtype, WHO grade 4

fig. S2. Single cell atlas of $CD45^{+}CD3^{-}$ cells in gliomas

(A) UMAP plot of $CD45^{+}CD3^{-}$ cells in gliomas demonstrating 14 clusters of myeloid lineage cells, two clusters of natural killer (NK) cells, one cluster of B cells, and one cluster of plasma cells. (B) Violin plots of expression level of canonical microglia genes across myeloid cell clusters. (C) Stacked bar plots highlighting the variation in proportion of myeloid cell populations among patients. (D) Box plots demonstrating the distribution of myeloid cell clusters across various classifications of gliomas. (E) Box plots showing no significant differences in the proportion of E-MDSC, M-MDSC, MAC2, and PMN-MDSC cell populations across common molecular variations in IDH-wildtype glioblastoma (i.e., EGFR amplification, MGMT methylation, and EGFR mutation)

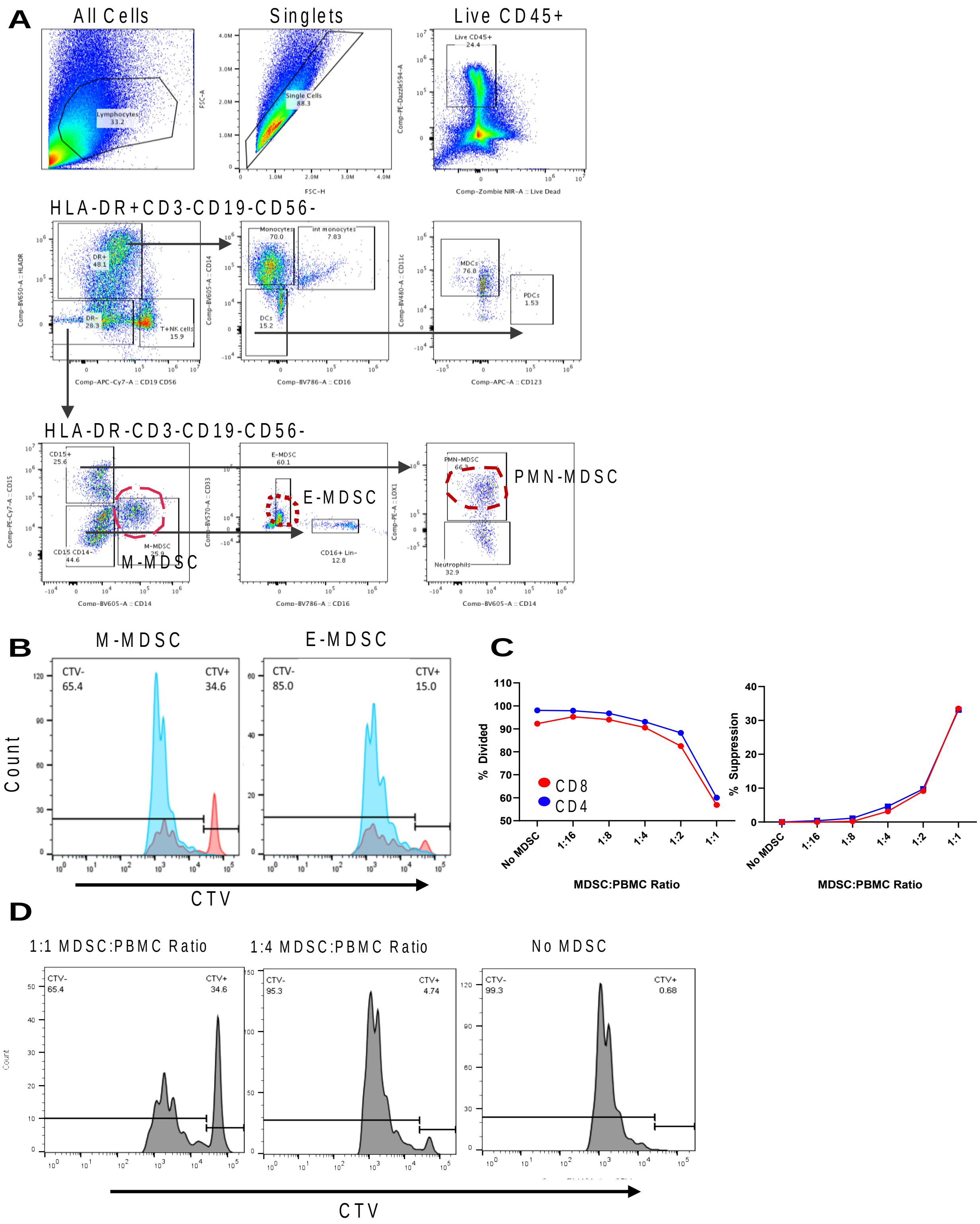
A
All Cells
Lymphocytes
33.2
Singlets
Single Cells
88.3
FSC-A
FSC-H
Live CD45+
Live CD45+
24.4
Comp-PE-Dazzle594-A
Comp-Zombie NIR-A :: Live Dead
HLA-DR+CD3-CD19-CD56-
DR+
48.1
DR-
28.3
T+NK cells
15.9
Comp-BV650-A :: HLADR
Comp-APC-Cy7-A :: CD19 CD56
Monocytes
70.0
int monocytes
7.83
DCs
15.2
Comp-BV605-A :: CD14
Comp-BV786-A :: CD16
MDCs
76.8
PDCs
1.53
Comp-BV480-A :: CD11c
Comp-APC-A :: CD123
HLA-DR-CD3-CD19-CD56-
CD15+
25.6
CD15 CD14-
44.6
M-MDSC
25.9
M-MDSC
Comp-PE-Cy7-A :: CD15
Comp-BV605-A :: CD14
E-MDSC
60.1
E-MDSC
CD16+ Lin-
12.8
Comp-BV570-A :: CD33
Comp-BV786-A :: CD16
PMN-MDSC
66.3
PMN-MDSC
Neutrophils
32.9
Comp-PE-A :: LOX1
Comp-BV605-A :: CD14
B
M-MDSC
E-MDSC
Count
CTV-
65.4
CTV+
34.6
CTV-
85.0
CTV+
15.0
CTV
C
% Divided
CD8
CD4
No MDSC
1:16
1:8
1:4
1:2
1:1
MDSC:PBMC Ratio
% Suppression
MDSC:PBMC Ratio
D
1:1 MDSC:PBMC Ratio
1:4 MDSC:PBMC Ratio
No MDSC
Count
CTV-
65.4
CTV+
34.6
CTV-
95.3
CTV+
4.74
CTV-
99.3
CTV+
0.68
CTV

fig. S3. E-MDSCs and M-MDSCs demonstrate T cell suppression

A) Multicolor flow cytometry gating strategy to isolate MDSC subsets E-MDSC (HLA-DR$^-$CD33$^+$CD14$^-$CD15$^-$CD16$^-$), M-MDSC (HLA-DR$^-$CD33$^+$CD14$^+$), PMN-MDSC (HLA-DR$^-$CD33$^+$CD15$^+$Lox1$^+$) from fresh single cell suspension of IDH-wildtype glioblastoma samples. (B) M-MDSC (left) and E-MDSC (right) were plated at a 1:1 ratio with healthy donor PBMC demonstrating suppression (red) when compared to PBMC cultured without MDSC (blue) as determined by differing cell trace violet (CTV) dilution. (C) M-MDSC suppression of CD4 and CD8 T cells quantified by percent divided and percent suppressed at varying M-MDSC:PBMC ratios. (D) M-MDSC suppression of PBMC demonstrated by M-MDSC:PBMC ratio titration from 1:1 (left), 1:4 (middle), and 0:1 (right) and CTV dilution.

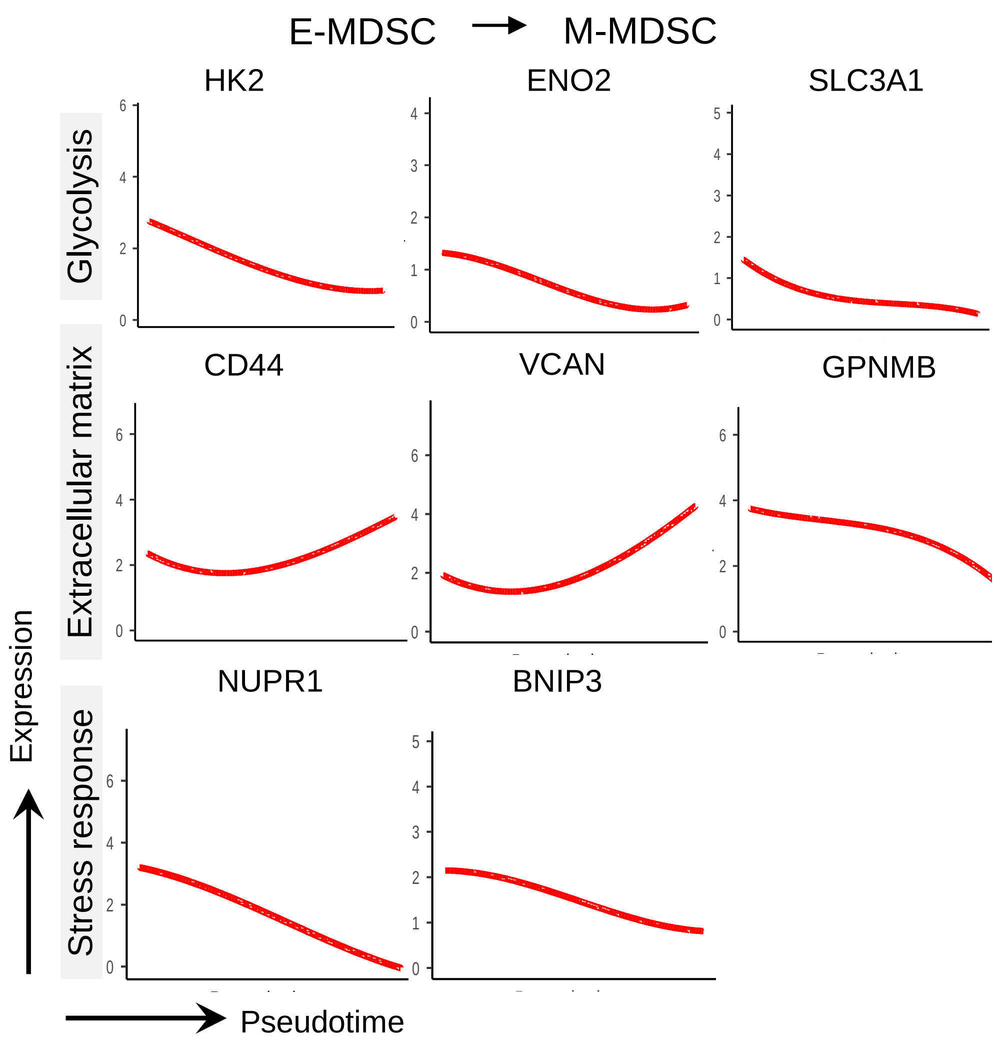

E-MDSC
M-MDSC
HK2
ENO2
SLC3A1
Glycolysis
CD44
VCAN
GPNMB
Extracellular matrix
NUPR1
BNIP3
Stress response
Expression
Pseudotime

fig. S4. E-MDSC and M-MDSC demonstrate temporal changes in select gene expression along transitional states.
Red curves represent the mean temporal function estimates of select genes along the E-MDSC to M-MDSC trajectory.

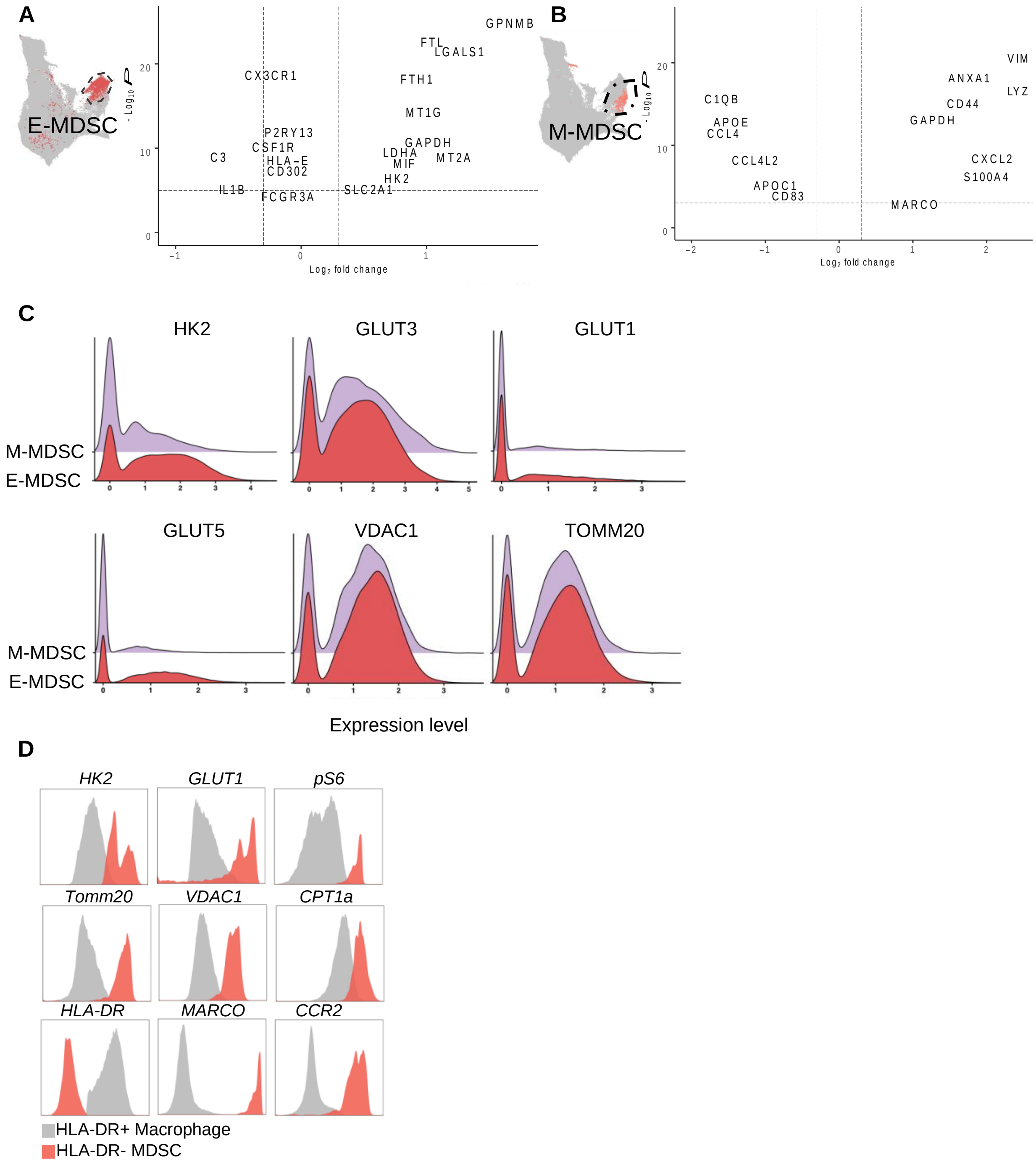
A
E-MDSC
GPNMB
FTL
LGALS1
CX3CR1
FTH1
MT1G
P2RY13
GAPDH
CSF1R
LDHA
C3
HLA-E
MIF
MT2A
CD302
HK2
IL1B
SLC2A1
FCGR3A
Log2 fold change
B
M-MDSC
VIM
ANXA1
LYZ
C1QB
CD44
APOE
GAPDH
CCL4
CXCL2
CCL4L2
S100A4
APOC1
CD83
MARCO
Log2 fold change
C
HK2
GLUT3
GLUT1
M-MDSC
E-MDSC
GLUT5
VDAC1
TOMM20
M-MDSC
E-MDSC
Expression level
D
HK2
GLUT1
pS6
Tomm20
VDAC1
CPT1a
HLA-DR
MARCO
CCR2
HLA-DR+ Macrophage
HLA-DR- MDSC

fig. S5. Distinct transcriptomic profiles of E-MDSC and M-MDSC populations.
(A) Volcano plot demonstrating upregulated and downregulated genes in E-MDSC relative to other myeloid populations (B) Volcano plot demonstrating upregulated and downregulated genes in M-MDSC relative to other myeloid populations. (C) Ridge plot demonstrating the distribution of expression of genes associated with metabolic pathways. (D) Histogram demonstrating increased expression of key proteins involved in metabolic pathways in HLA-DR low MDSC populations compared to HLA-DR high macrophage populations.

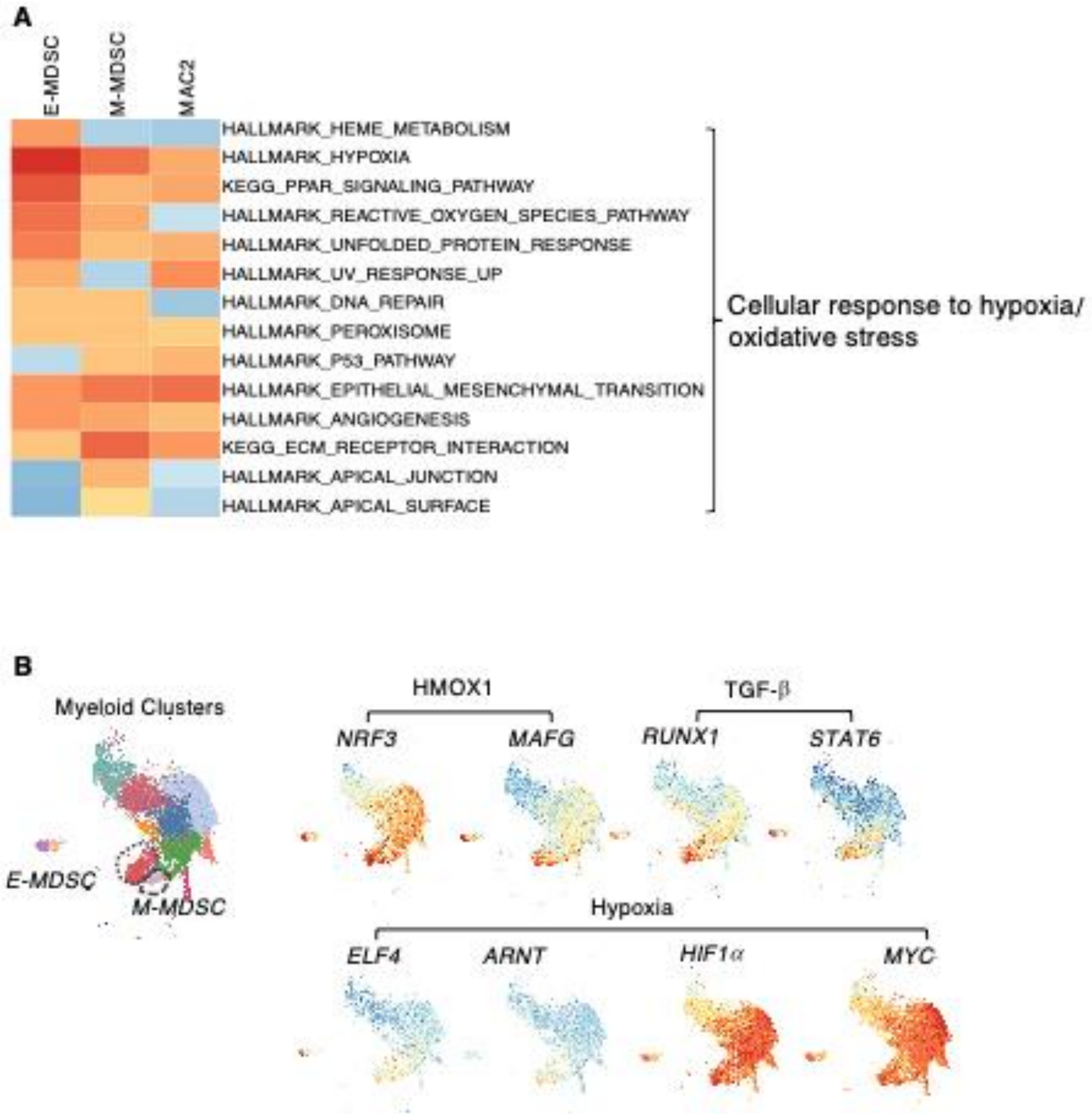
A
E-MDSC
M-MDSC
MAC2
HALLMARK_HEME_METABOLISM
HALLMARK_HYPOXIA
KEGG_PPAR_SIGNALING_PATHWAY
HALLMARK_REACTIVE_OXYGEN_SPECIES_PATHWAY
HALLMARK_UNFOLDED_PROTEIN_RESPONSE
HALLMARK_UV_RESPONSE_UP
HALLMARK_DNA_REPAIR
HALLMARK_PEROXISOME
HALLMARK_P53_PATHWAY
HALLMARK_EPITHELIAL_MESENCHYMAL_TRANSITION
HALLMARK_ANGIOGENESIS
KEGG_ECM_RECEPTOR_INTERACTION
HALLMARK_APICAL_JUNCTION
HALLMARK_APICAL_SURFACE
Cellular response to hypoxia/ oxidative stress
B
Myeloid Clusters
E-MDSC
M-MDSC
HMOX1
NRF3
MAFG
TGF-β
RUNX1
STAT6
Hypoxia
ELF4
ARNT
HIF1α
MYC

fig. S6. M-MDSC and E-MDSC demonstrate upregulation of hypoxia and cellular stress response pathways

(A) Heatmap of Hallmark and KEGG GSEA enriched pathways among E-MDSC, M-MDSC, and MAC2. Enlarged panel demonstrating top upregulated pathways in cellular response to stress and tumorigenesis present in MDSCs. (B) UMAP plots demonstrating increased activation of transcription factors that regulate antioxidant pathways, hypoxia, and cellular response to stress.

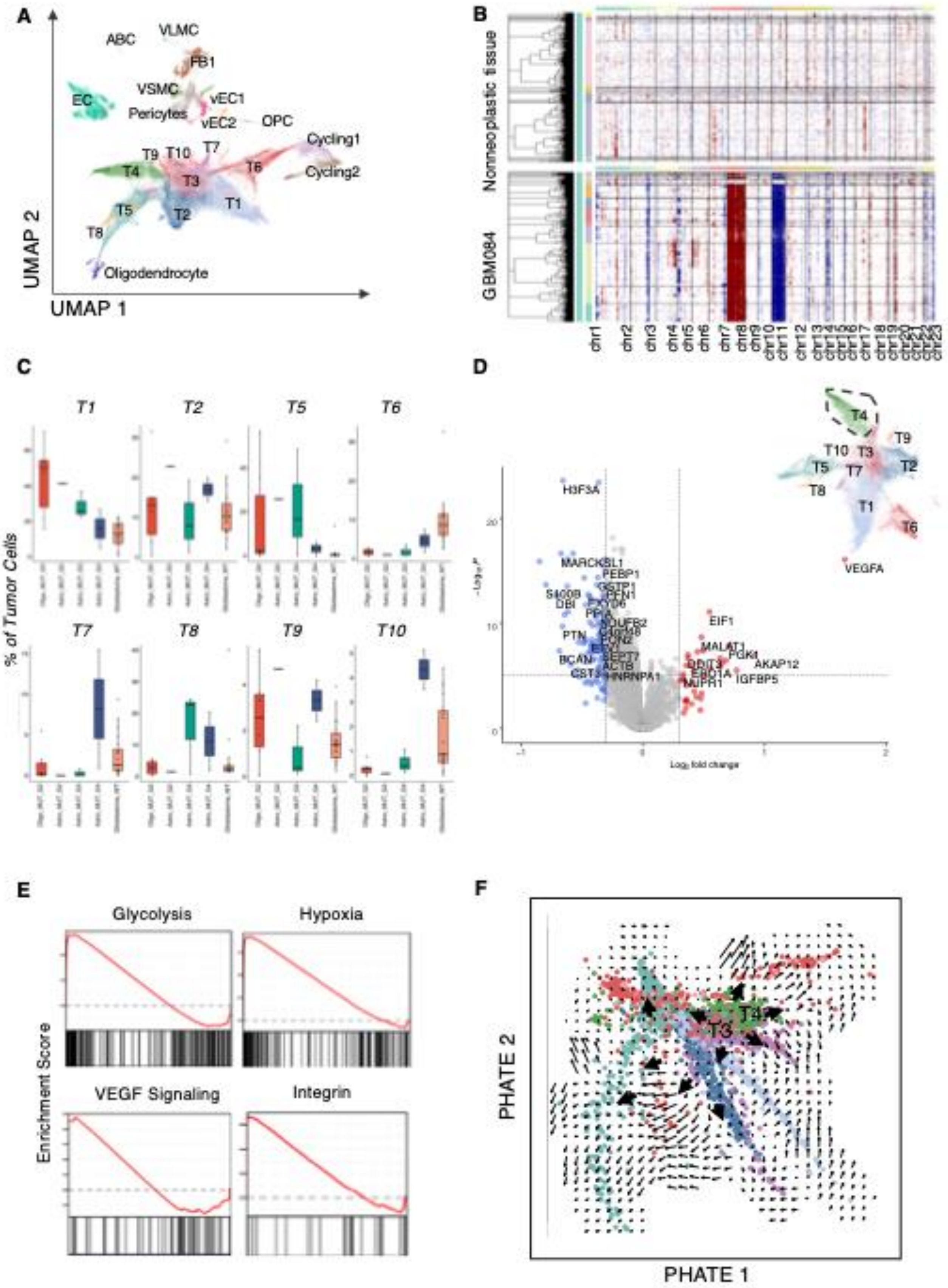
A
ABC
VLMC
FB1
EC
VSMC
vEC1
Pericytes
vEC2
OPC
Cycling1
T9
T10
T7
T6
Cycling2
T4
T3
T1
T5
T2
T8
Oligodendrocyte
UMAP 2
UMAP 1
B
Nonneoplastic tissue
GBM084
chr1 chr2 chr3 chr4 chr5 chr6 chr7 chr8 chr9 chr10 chr11 chr12 chr13 chr14 chr15 chr16 chr17 chr18 chr19 chr20 chr21 chr22 chr23
C
T1
T2
T5
T6
T7
T8
T9
T10
% of Tumor Cells
D
T4
T10
T3
T9
T5
T7
T2
T8
T1
T6
VEGFA
H3F3A
MARCKSL1
PEBP1
GSTP1
S100B
PFN1
DBI
FXYD6
PPIA
NDUFB2
PTN
BCAN
CST3
SEPT7
ACTB
HNRNPA1
EIF1
MALAT1
PGK1
AKAP12
DDIT3
IGFBP5
Log₂ fold change
E
Glycolysis
Hypoxia
VEGF Signaling
Integrin
Enrichment Score
F
T4
T3
PHATE 2
PHATE 1

fig. S7. Single cell atlas of CD45$^-$ cells in gliomas

(A) UMAP plot of CD45$^-$ cells in gliomas demonstrating 12 clusters of neoplastic cells and 12 clusters of stromal cells. (B) Heatmap demonstrating large-scale CNVs for individual cells (rows) across each cluster of CD45$^-$ cells from a representative tumor patient (bottom, GBM084), and representative nonneoplastic tissue (top, GBM076) inferred based on the average expression of 100 genes surrounding each chromosomal position (column). Malignant cell clusters exhibit chromosome 7 gain (red) and chromosome 10 loss (blue), which are characteristic of glioblastoma. (C) Box plots showing proportions of tumor clusters across various subtypes of gliomas. (D) Volcano plot demonstrating upregulated and downregulated genes expressed by T4 relative to other tumor populations. (E) Gene set enrichment scores of gene sets enriched in T4 demonstrating upregulation of metabolic, hypoxia, and angiogenesis pathways. (F) RNA velocities visualized on a phate map projection of tumor cells in IDH-wildtype glioblastoma.

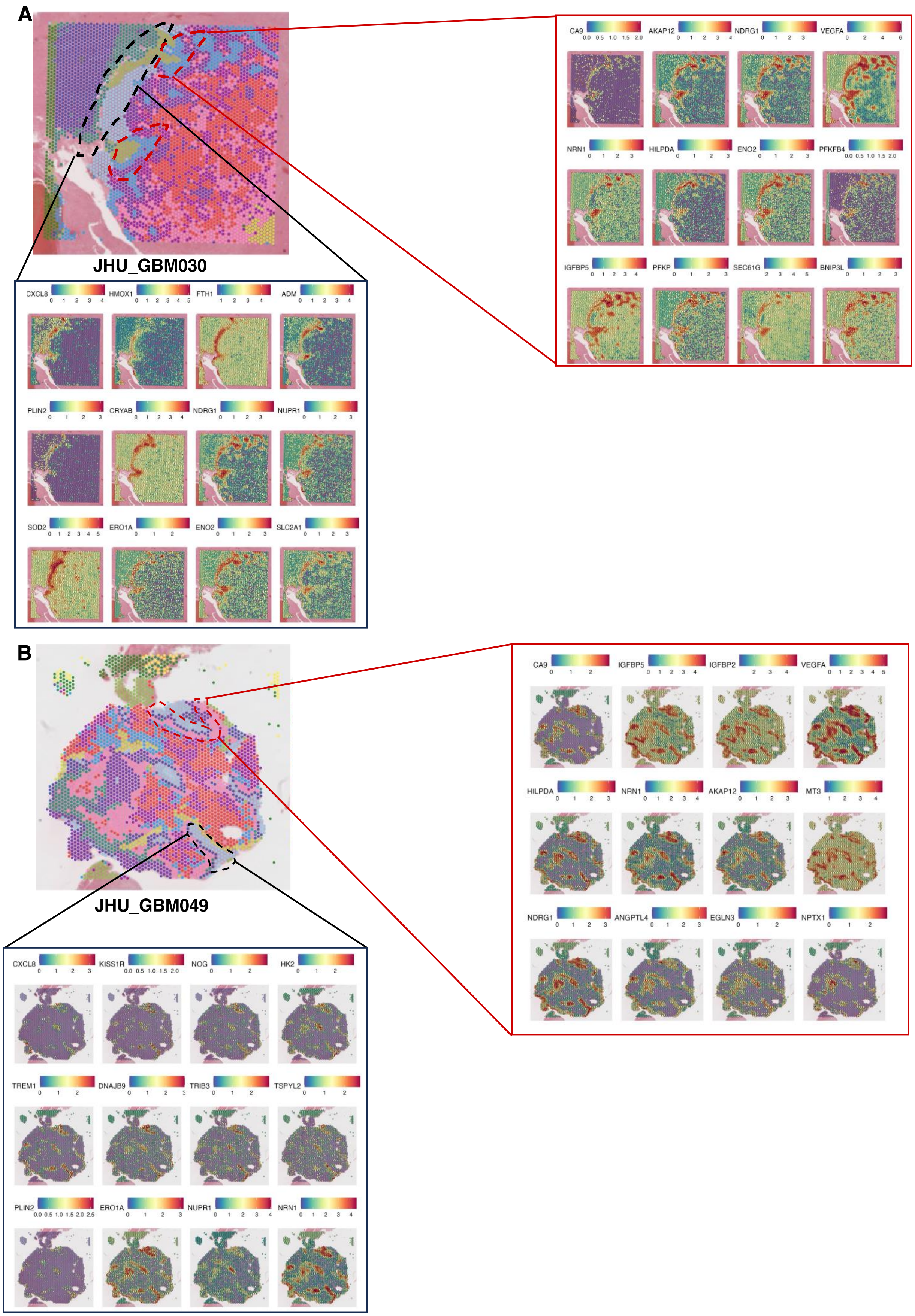
A
JHU_GBM030
CA9
AKAP12
NDRG1
VEGFA
NRN1
HILPDA
ENO2
PFKFB4
IGFBP5
PFKP
SEC61G
BNIP3L
CXCL8
HMOX1
FTH1
ADM
PLIN2
CRYAB
NDRG1
NUPR1
SOD2
ERO1A
ENO2
SLC2A1
B
JHU_GBM049
CA9
IGFBP5
IGFBP2
VEGFA
HILPDA
NRN1
AKAP12
MT3
NDRG1
ANGPTL4
EGLN3
NPTX1
CXCL8
KISS1R
NOG
HK2
TREM1
DNAJB9
TRIB3
TSPYL2
PLIN2
ERO1A
NUPR1
NRN1

fig. S8. Pseudopalisading region of IDH-wildtype glioblastoma demonstrates upregulation of representative genes found in MDSCs and T4 tumor cells

(A, B) Spatial transcriptomic analysis demonstrating recapitulation of scRNA-seq clusters with differential gene expressions similar to E-MDSC and T4 populations co-localized to the pseudopalisading region.

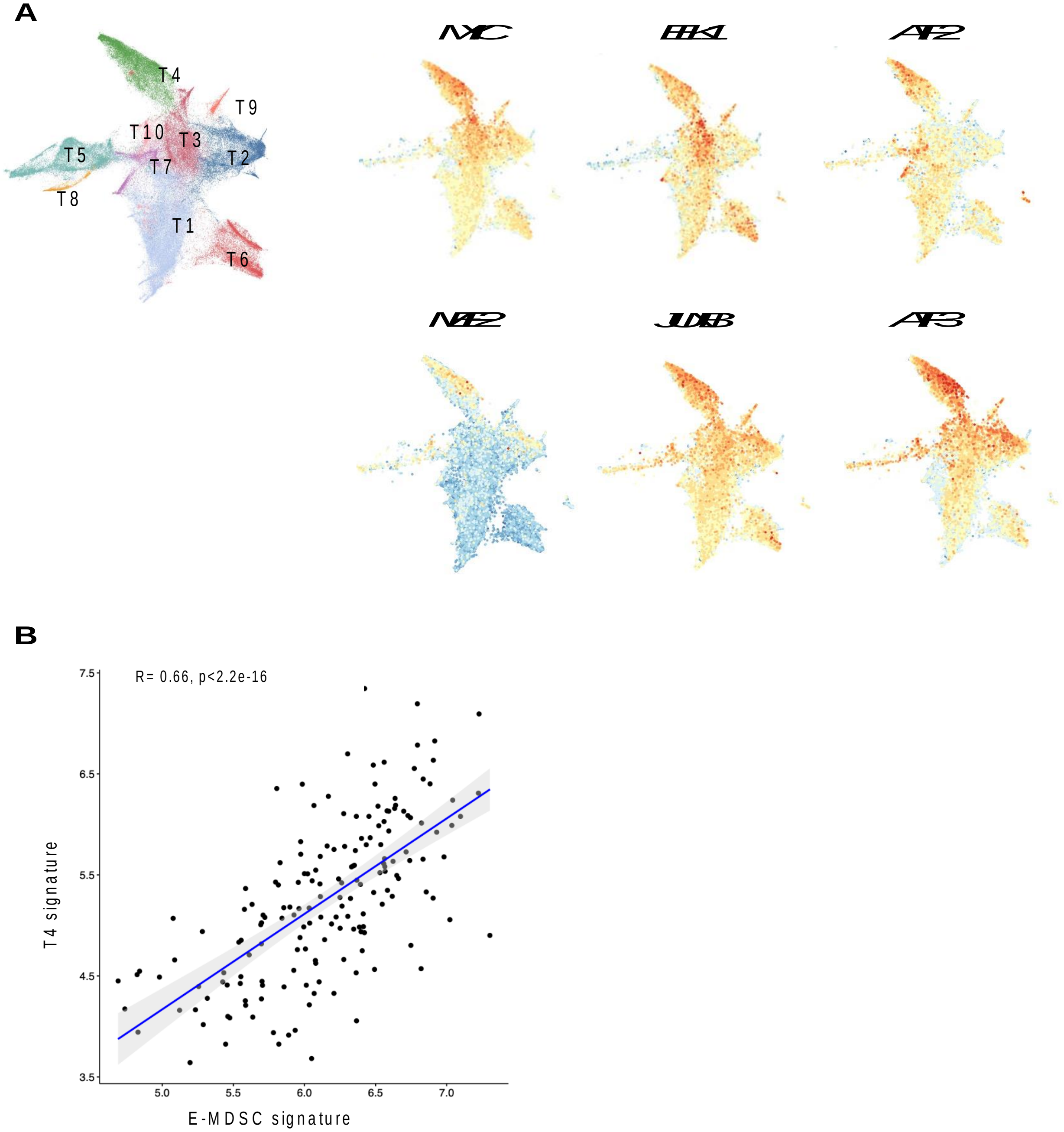
A
T4
T9
T10
T3
T5
T7
T2
T8
T1
T6
B
R= 0.66, p<2.2e-16
7.5
6.5
5.5
4.5
3.5
T4 signature
5.0
5.5
6.0
6.5
7.0
E-MDSC signature

fig. S9: T4 glioma cells demonstrate transcription factor activation associated with growth factor and angiogenesis

(A) UMAP plots demonstrating activation scores of transcription factors associated with growth factor and angiogenesis signaling pathways preferentially activated in glioma tumor cluster T4 with stem-like and mesenchymal programs. (B) Correlation of E-MDSC and T4 signatures based on TCGA high grade glioma data. Each dot represents a patient (Spearman correlation).

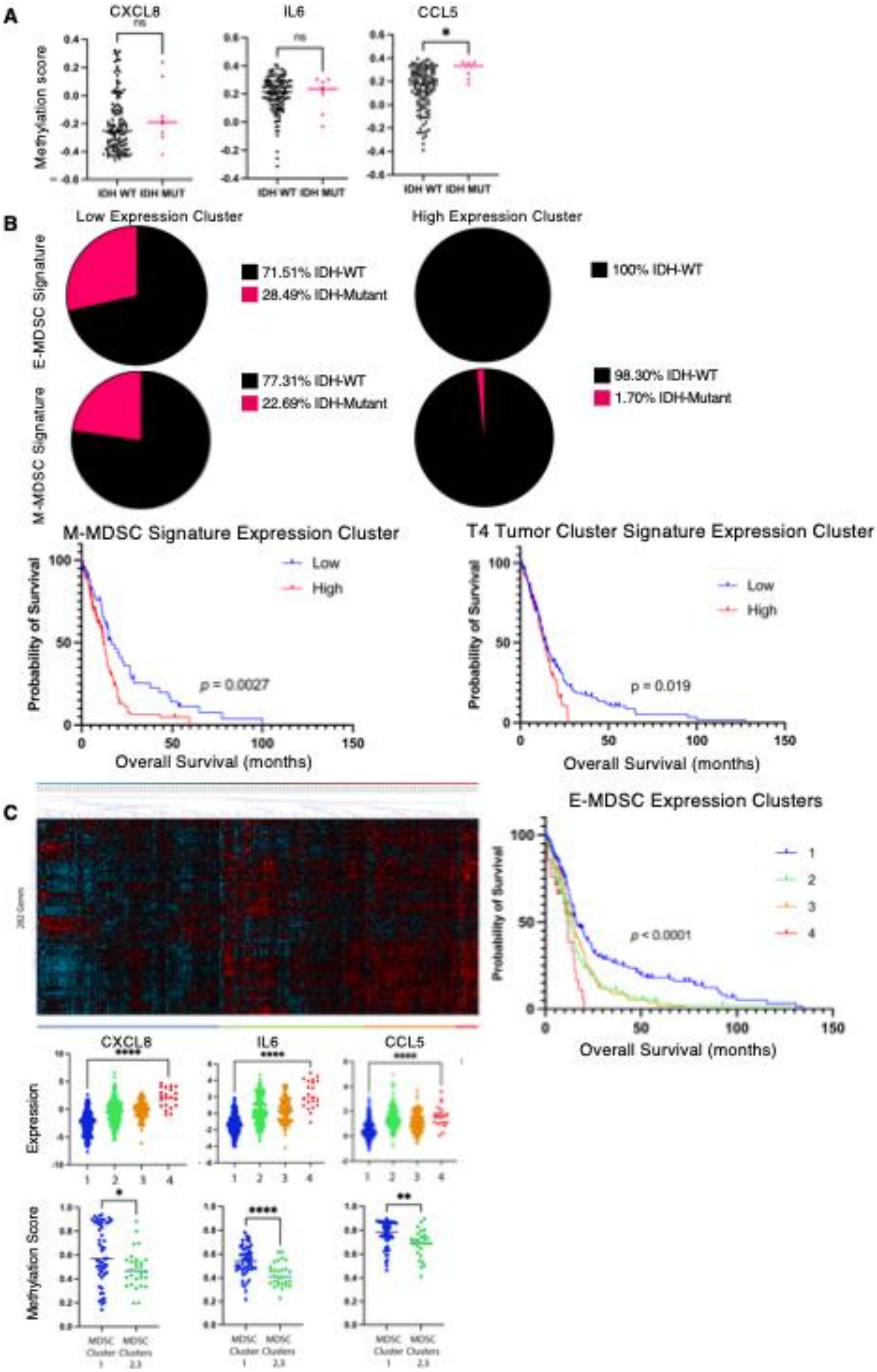

A
CXCL8
IL6
CCL5
Methylation score
IDH WT IDH MUT
B
Low Expression Cluster
High Expression Cluster
E-MDSC Signature
M-MDSC Signature
71.51% IDH-WT
28.49% IDH-Mutant
100% IDH-WT
77.31% IDH-WT
22.69% IDH-Mutant
98.30% IDH-WT
1.70% IDH-Mutant
M-MDSC Signature Expression Cluster
T4 Tumor Cluster Signature Expression Cluster
Low
High
Probability of Survival
Overall Survival (months)
p = 0.0027
p = 0.019
C
E-MDSC Expression Clusters
p < 0.0001
Expression
Methylation Score
MDSC Cluster 1
MDSC Clusters 2,3

fig. S10. MDSC and T4 gene expression signature stratifies glioblastoma patient survival
(A) Dot plots demonstrating decreased methylation score of MDSC recruiting and activating chemokines and cytokines in IDH-wildtype grade 4 glioblastomas compared with IDH-mutant grade 4 astrocytomas. (B) Pie chart demonstrating that high expressing group of E-MDSC and M-MDSC signatures consist of 100% and 98.30% IDH-wildtype glioblastoma patients, respectively, while the low expressing group consist of a mixture of IDH-wildtype and IDH-mutant tumors. Kaplan Meier curve illustrating that the M-MDSC and T4 expression signature stratified IDH-wildtype glioblastoma patient survival (C) Heatmap and Kaplan Meier curve demonstrating clustering of TCGA glioma samples into four clusters based on expression levels of E-MDSC signature with stratification by survival. Dot plots demonstrating decreased methylation score and increased expression of MDSC recruiting and activating chemokines in patients with high representation of E-MDSC signature.

Table S1. Patient clinical characteristic and sequencing statistics

Patient ID	Age/Sex	2021 Classification	WHO Grade	CD45+CD3+	CD45+CD3-	CD45-
GBM006	67/F	Glioblastoma, IDH-WT	4	3652	6841	3027
GBM009	70/M	Glioblastoma, IDH-WT	4	2912	572	2246
GBM010	53/M	Glioblastoma, IDH-WT	4	5829	1883	1945
GBM029	74/F	Glioblastoma, IDH-WT	4	2805	885	1332
GBM030	66/M	Glioblastoma, IDH-WT	4	5546	1965	1734
GBM035	66/F	Glioblastoma, IDH-WT	4	2415	1993	2243
GBM036	61/F	Glioblastoma, IDH-WT	4	6800	2087	8312
GBM037	73/F	Glioblastoma, IDH-WT	4	6375	2209	6310
GBM043	63/M	Glioblastoma, IDH-WT	4	7102	4249	7706
GBM045	44/F	Oligo, IDH-mutant & 1p/19q codel.	2	13018	3795	606
GBM046	64/M	Glioblastoma, IDH-WT	4	1735	3437	0
GBM048	56/F	Glioblastoma, IDH-WT	4	11500	4767	7220
GBM049	71/M	Glioblastoma, IDH-WT	4	11423	3973	12796
GBM050	64/F	Glioblastoma, IDH-WT	4	12818	7502	12837
GBM051	66/M	Glioblastoma, IDH-WT	4	21456	8879	7592
GBM052	40/M	Glioblastoma, IDH-WT	4	15090	5515	9924
GBM054	64/F	Glioblastoma, IDH-WT	4	9542	6261	10049
GBM055	37/F	Astrocytoma, IDH-mutant	3	15431	8269	5925
GBM056	78/F	Glioblastoma, IDH-WT	4	12700	6848	9758
GBM057	47/F	Glioblastoma, IDH-WT	4	17583	9955	14802
GBM059	38/F	Oligo, IDH-mutant & 1p/19q codel.	2	7432	4875	8021
GBM060	31/F	Astrocytoma, IDH-mutant	3	4776	9234	9922
GBM064	28/M	Astrocytoma, IDH-mutant	4	12668	6749	10067
GBM065	77/M	Glioblastoma, IDH-WT	4	14104	5516	7451
GBM066	55/M	Glioblastoma, IDH-WT	4	17726	3563	8999
GBM068	42/F	Oligo, IDH-mutant & 1p/19q codel.	2	4280	13037	10448
GBM069	66/F	Oligo, IDH-mutant & 1p/19q codel.	2	11583	5450	5030
GBM070	32/F	Astrocytoma, IDH-mutant	2	2790	3993	10345
GBM073	33/M	Astrocytoma, IDH-mutant	4	15246	22129	11674
GBM074	64/F	Oligo, IDH-mutant & 1p/19q codel.	2	17912	5004	11092
GBM075	31/F	Astrocytoma, IDH-mutant	3	11250	2495	1880
GBM076	22/M	NA	Non-neoplastic	23344	10284	9529
GBM077	33/M	NA	Non-neoplastic	13914	6472	14279
GBM081	24/F	Oligo, IDH-mutant & 1p/19q codel.	2	6819	7800	10153
GBM082	83/F	Glioblastoma, IDH-WT	4	5596	11960	9180
GBM086	31/F	NA	Non-neoplastic	1761	3444	597
GBM089	26/M	NA	Non-neoplastic	4887	6787	5697
GBM090	57/M	NA	Non-neoplastic	17765	11471	19389
GBM094	83/F	NA	Non-neoplastic	2027	8045	5266

Oligo: oligodendroglioma, codel: codeleted, WT: wildtype

Table S2. Differentially expressed genes for scRNAseq myeloid clusters.

Table S3. Immunofluorescence panel for sorting of MDSC subsets

Fluorophore	Marker	Manufacturer	Catalog #
L/D	PI	Invitrogen	BMS500PI
CD45	FITC	Biolegend	368507
CD3	APC-Cy7	Biolegend	344818
CD19	APC-Cy7	Biolegend	363010
CD56	APC-Cy7	Biolegend	362512
HLA-DR	BB700	BD	566480
CD33	BV785	Biolegend	303427
CD14	BV605	Biolegend	301834
CD15	PE-Cy7	Biolegend	301924
CD16	BV711	Biolegend	302043

Table S4. Immunofluorescence panel for protein validation of MDSC metabolic pathways

**Fluorophore**	**Marker**	**Manufacturer**	**Catalog #**
L/D	Zombie NIR	Invitrogen	L34975
CD45	Spark NIR	Biolegend	368552
CD11c	BV480	Biolegend	566135
CD3	APC-Cy7	Biolegend	344818
CD19	APC-Cy7	Biolegend	363010
CD56	APC-Cy7	Biolegend	362512
HLA-DR	BV750	Biolegend	307672
CD33	BV570	Biolegend	303417
CD14	BV605	Biolegend	301834
CD15	PE-Cy7	Biolegend	301924
CD16	BV786	Biolegend	302046
CCR2	BV510	Biolegend	357218
LOX1	PE	Biolegend	358604
CD163	BV650	Biolegend	333647
CD206	BV711	Biolegend	321136
MARCO	APC	eBioscience	17-544-741
HK2	Alexa 680	Abcam	ab210047
VDAC1	AF532	Abcam	ab14734
Tomm20	AF405	Abcam	ab210047
GLUT1	AF647	Abcam	ab195020
CPT1a	AF488	Abcam	ab171449
pS6	AF594	Abcam	ab236224

Table S5. Ligand-receptor interaction pairs used to perform cluster interaction analysis

Table S6. Gene sets utilized from Molecular Signatures Database
